# Absence of complementary sex determination in two *Leptopilina* species (Figitidae, Hymenoptera) and a reconsideration of its incompatibility with endosymbiont‐induced thelytoky

**DOI:** 10.1111/1744-7917.12969

**Published:** 2021-10-25

**Authors:** Fangying Chen, Martijn Schenkel, Elzemiek Geuverink, Louis van de Zande, Leo W. Beukeboom

**Affiliations:** ^1^ Groningen Institute for Evolutionary Life Sciences University of Groningen P.O. Box 11103 Groningen 9700 CC the Netherlands

**Keywords:** arrhenotoky, haplodiploidy, inbreeding, sex determination, thelytoky, *Wolbachia* bacteria

## Abstract

Complementary sex determination (CSD) is a widespread sex determination mechanism in haplodiploid Hymenoptera. Under CSD, sex is determined by the allelic state of one or multiple CSD loci. Heterozygosity at one or more loci leads to female development, whereas hemizygosity of haploid eggs and homozygosity of diploid eggs results in male development. Sexual (arrhenotokous) reproduction normally yields haploid male and diploid female offspring. Under asexual reproduction (thelytoky), diploidized unfertilized eggs develop into females. Thelytoky is often induced by bacterial endosymbionts that achieve egg diploidization by gamete duplication. As gamete duplication leads to complete homozygosity, endosymbiont‐induced thelytokous reproduction is presumed to be incompatible with CSD, which relies on heterozygosity for female development. Previously, we excluded CSD in four *Asobara* (Braconidae) species and proposed a two‐step mechanism for *Wolbachia‐*induced thelytoky in *Asobara japonica*. Here, we conclusively reject CSD in two cynipid wasp species, *Leptopilina heterotoma* and *Leptopilina clavipes*. We further show that thelytoky in *L. clavipes* depends on *Wolbachia* titer but that diploidization and feminization steps cannot be separated, unlike in *A. japonica*. We discuss what these results reveal about the sex determination mechanism of *L. clavipes* and the presumed incompatibility between CSD and endosymbiont‐induced thelytoky in the Hymenoptera.

## Graphical abstract

Under complementary sex determination (CSD), if the fertilized eggs are homozygous, they develop into diploid males. Forms of thelytoky induced by endosymbiont lead to genome homozygosity as well. By presenting our results in two cynipid wasp species *Leptopilina heterotoma* and *Leptopilina clavipes*, we discuss the presumed incompatibility between CSD and endosymbiont‐induced thelytoky in Hymenoptera.

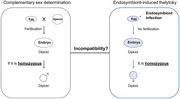

## Introduction

Hymenopteran insects have sexual haplodiploid reproduction, termed arrhenotoky. Diploid females develop from fertilized eggs and inherit one chromosome set from each parent, whereas males arise from unfertilized eggs and carry only one genome set, inherited from their mother. Some hymenopterans reproduce asexually, by thelytoky, under which females develop from unfertilized diploid eggs. Thelytoky can be genetically determined but in many cases is induced by infectious bacterial endosymbionts, such as *Wolbachia*, *Cardinium* and *Rickettsia* (reviewed in Stouthamer, 1997; Heimpel & de Boer, 2008; Ma & Schwander, 2017). Such endosymbionts can thus interfere with the reproductive system and development of their host.

In many hymenopterans, sex is determined by a complementary sex determination (CSD) mechanism, and this mechanism has been postulated as being the ancestral state of hymenopteran reproduction (Whiting, 1943; Cook, 1993a; Beye *et al*., 2003; van Wilgenburg *et al*., 2006; Heimpel & de Boer, 2008; Asplen *et al*., 2009). Under CSD, sex is determined by the individual's genotype for one or more CSD loci. Heterozygosity of CSD loci results in female development whereas homozygosity or hemizygosity leads to diploid and haploid males, respectively. CSD can involve only one locus, called single‐locus CSD (sl‐CSD), but also multiple loci (ml‐CSD) (Crozier, 1971; Cook, 1993a; de Boer *et al*., 2008; Harpur *et al*., 2013; Miyakawa & Mikheyev, 2015; Paladino *et al*., 2015). Under ml‐CSD, heterozygosity for at least one CSD locus is required for female development. Hemizygosity or homozygosity at all CSD loci leads to male development. For the honeybee (*Apis mellifera*) an sl‐CSD locus has been identified (Beye *et al*., 2003) and this *csd* gene is a duplication (paralog) of feminizer, the *A. mellifera* ortholog of transformer (*tra*) (Hasselmann *et al*., 2008). No ml‐CSD loci have been characterized (but see Matthey‐Doret *et al*., 2019 for an attempt).

The available data on the phylogenetic distribution of CSD within the Hymenoptera show that it occurs widely among the Apoidea (ants, wasps and bees), but appears to be largely absent in most Parasitica (parasitoid wasp) groups, whereas several more basal lineages have not been investigated (van Wilgenburg *et al*., 2006; Heimpel & de Boer, 2008; Asplen *et al*., 2009; Harpur *et al*., 2013). The ancestral state reconstruction by Asplen *et al*. (2009) is based upon two important criteria for rejecting CSD: (i) inbreeding does not increase progeny sex ratio or diploid male production; and (ii) presence of endosymbiont‐induced thelytoky, as this often involves genome homozygosity by gamete duplication (Suomalainen *et al*., 1987; Pannebakker *et al*., 2002; Mateo Leach *et al*., 2009). CSD is considered mechanistically incompatible with gamete duplication because homozygosity of diploid eggs would result in diploid males rather than females (Cook, 1993b; Stouthamer & Kazmer, 1994; van Wilgenburg *et al*., 2006). The reconstruction led Asplen *et al*. (2009) to predict that species from the Parasitica families Chalcidoidea, Cynipoidea and Platygastroidea would not have any form of CSD, whereas for three species of the Braconidae, that have been shown to lack sl‐CSD, ml‐CSD is inferred. However, ml‐CSD was ruled out for *Asobara tabida* and three additional *Asobara* species by Ma *et al*. (2013). In addition, *Wolbachia‐*induced thelytoky is present in *Asobara japonica* (Kremer *et al*., 2009). These results are at odds with the prediction of CSD presence in Braconidae by Asplen *et al*. (2009). Here, we present data that might explain this apparent paradox.

Ma *et al*. (2015) proposed a two‐step model for *Wolbachia‐*induced thelytoky in *A. japonica*. The two separate steps, diploidization of the unfertilized egg and feminization of the diploidized embryo, are separate events, and require a different *Wolbachia* titer. Gradually lowering the *Wolbachia* content in thelytokous *A. japonica* females caused them to initially produce diploid males, and after complete removal of the endosymbiont, only haploid male offspring. These results indicate that the *Wolbachia* endosymbiont can overrule the sex determination of its host and such active feminization of diploid embryos may be independent of their genotype. This observation in turn challenges the incompatibility of CSD and forms of thelytoky that lead to genome homozygosity.

To gain more insight in the interconnection of host sex determination and endosymbiont‐induced thelytoky, we tested the cynipid wasps *Leptopilina heterotoma* and *Leptopilina clavipes* (superfamily Cynipoidea) for the presence of CSD using inbreeding crosses. *L. heterotoma* only reproduces by arrhenotoky, but for *L. clavipes*, both arrhenotokous and *Wolbachia‐*infected thelytokous populations are known (Pannebakker *et al*., 2004b; Kraaijeveld *et al*., 2011). The underlying cytological mechanism for diploidization in thelytokous *L. clavipes* is gamete duplication (Pannebakker *et al*., 2004a). We investigated whether *Wolbachia* also actively feminizes the resulting diploid embryos using antibiotic treatment of infected thelytokous females. We discuss our results in combination with those obtained earlier by Ma *et al*. (2013, 2015) to reconsider the proposed incompatibility of CSD and *Wolbachia*‐induced thelytoky in Hymenoptera.

## Materials and methods

### Strains and culture conditions

Two arrhenotokous laboratory strains of both *L. heterotoma* and *L. clavipes* were used. *L. heterotoma* strain Santa Christina (SC) was collected from Santa Christina d'Aro, Spain (41.80775°N, 3.003383°E) in 2015 and strain Vosbergen (VB) from Vosbergen, the Netherlands (53.137066°N, 6.594072°E) in 2015. The sexual *L. clavipes* strains Calonge 1 (CA1) and El Pou del Glac 2 (EPG2) were collected from Calonge, Spain at separate sites (41.877063°N, 3.077668°E and 41.931167°N, 3.028833°E, respectively) in 2017. Prior to testing, the *L. heterotoma* strains had been maintained in the laboratory for approximately 13 generations and *L. clavipes* for 11 generations with approximately 50 individuals each generation. They were maintained and tested on *Drosophila melanogaster* larvae as hosts at 25 °C under constant light.

The thelytokous *L. clavipes* strain Losser (LS) was collected from Losser, the Netherlands (52.279290°N, 6.958964°E) in 2017 and was maintained on *Drosophila virilis* larvae at 25 °C under constant light.

### Inbreeding experiments

Under CSD, inbreeding leads to an increase of the proportion of male progeny as a result of increased homozygosity. Hence, inbreeding crosses have been used for many species to determine the presence or absence of CSD (reviewed in Cook, 1993b; van Wilgenburg *et al*., 2006; Heimpel & de Boer, 2008; Harpur *et al*., 2013). Increasing the inbreeding coefficient over consecutive generations increases the chance of homozygosity at CSD loci and thus diploid male production (e.g. de Boer *et al*., 2008). The progeny sex ratio further depends on the viability of diploid males, the fertilization rate and the number of sex determination loci (Table 1). Therefore, a protocol of multiple inbreeding generations must be used to assess ml‐CSD (Cook, 1993a; Beukeboom *et al*., 2000; Ma *et al*., 2013; Paladino *et al*., 2015; Liu *et al*., 2019).

**Table 1:** Predicted proportions of diploid females, haploid and diploid males, and progeny sex ratio, under different CSD scenarios

	No CSD	CSD with inviable diploid male	CSD with viable diploid male
Proportion female (F)	f	f‐i	(1‐h)f
Proportion haploid male (M)	1‐f	1‐f	1‐f
Proportion diploid male (D)	0	0	hf
Sex ratio	M / (F + M) or (1‐f) / ([1‐f]+f)	M / (F + M)^†^ or (1‐f) / ([f‐i] + [1‐f])	(M + D) / (F + M + D) or ([1‐f] + hf) / ([1‐h]f + [1‐f] + hf)

^†^ Note that the proportion females will be lower because a fraction of fertilized eggs will yield inviable diploid males, i.e. f = f‐i

f = fertilization rate, i = proportion inviable diploid males and h = chance of homozygosity at all complementary sex determination (CSD) loci.

For *L. heterotoma*, experimental strains were established by individually mating 20 virgin females from one strain with 20 males of the other strain in both directions. The aim was to increase genetic variation of the starting lines prior to inbreeding to maximize the heterozygosity for any potential CSD loci. F1 virgin females of both reciprocal crosses were collected and provided with host larvae for parasitization at 25 °C for 1–2 d, after which they were stored at 12 °C for 3 weeks until their sons had emerged. The females were then split into an experimental and control group: each female from the experimental group was placed with one of her sons for 12–24 h to mate (mother–son or M‐S cross), and the control females were individually mated with one non‐related male from the mixed stock culture (Fig. S1). Virgin females descending from the M‐S cross were then split into an experimental and control group as well. Next, experimental females were individually mated with one of their brothers (brother–sister or B‐S cross), and control females were individually mated with a randomly picked male from the mixed stock (Fig. S1). This was repeated for six generations (B‐S 1 to B‐S 6). In a first experiment for *L. heterotoma*, no control group was established for the M‐S cross, so only B‐S 1 to B‐S 6 have a non‐inbred control. In a second experiment, four generations of B‐S crosses following an M‐S cross were set up with controls for every cross.

As *L. clavipes* females are reluctant to mate after cold storage, an initial M‐S cross could not be established. Instead, the first of six generations of inbreeding consisted of a B‐S cross (B‐S 1, Fig. S2). The remainder of the procedure was similar to that of *L. heterotoma*. In every generation, the number of emerged male and female wasps (sex ratio = proportion male) was recorded for both species.

### Progeny sex ratios and detection of diploid males

Inbreeding under CSD causes a fraction of fertilized eggs to develop into diploid males rather than diploid females. All‐male broods may have two causes; they are either the result of unmated females or of homozygosity of all fertilized eggs by chance. Under sl‐CSD, a maximum of 50% of fertilized eggs are expected to become homozygous, and this proportion decreases with an increasing number of CSD loci, so that the chance of all‐male broods as a result of homozygosity of all fertilized eggs decreases with brood size.

The occurrence of diploid males further depends on whether they are viable or not. *L. heterotoma* and *L. clavipes* are solitary species meaning that a single adult wasp emerges from a successfully parasitized host. To account for possible production of inviable diploid males, the number of emerged *Drosophila* flies and the number of non‐emerged *Drosophila* pupae were counted, as they may indicate premature wasp death at the egg or pupal stage. Non‐emerged host pupae were sometimes opened to inspect them for non‐emerged wasps, whereby the sex of non‐emerged wasps was determined based on antennal morphology.

A representative subset of male offspring was tested for ploidy by flow cytometry as described in de Boer *et al*. (2007). In short, wasp heads were homogenized in 50 *μ*L Galbraith buffer and stained with 10 *μ*L propidium iodide (2.5 mg/mL). DNA content was measured with a Coulter Epics MXL flow cytometer (Beckman Coulter) or a MACSQuant Analyzer 10 (Miltenyi Biotec) and analyzed with MACSQuantify™ Software (Miltenyi Biotec). Males were classified as haploid or diploid based on reference individuals of known ploidy that were analyzed at the same time. In *L. heterotoma*, individuals from B‐S 3 were tested for ploidy, and in *L. clavipes*, individuals from B‐S 5 and B‐S 6.

### Simulations

An individual‐based model adapted from Cook (1993a), de Boer *et al*. (2012, 2008) and Ma *et al*. (2013) was used to predict the sex ratio and diploid male ratio under different CSD conditions (i.e. number of CSD loci). Simulations were performed assuming 1, 2, 5 and 10 putative CSD loci. Diploid individuals developed as female if they were heterozygous for one or more CSD loci, but as male if they were homozygous for all loci; all haploid (hemizygous) individuals developed as males. For direct comparison of the simulated and observed data, we randomly sampled the brood sizes and fertilization proportions from females in the entire control pedigree. All simulations were initiated with an F0 outcross between a fully heterozygous female (e.g. with a genotype A/B for all CSD loci) and an unrelated haploid male (with a C allele for all CSD loci) so that all diploid offspring were heterozygous (A/C or B/C) for each of the CSD loci; haploid males generated in this cross naturally had a hemizygous A or B genotype for each of the CSD loci. After this outcrossing, we simulated a series of inbreeding crosses. For *L. heterotoma*, the first inbreeding cross was an M‐S cross followed by B‐S crosses, whereas for *L. clavipes* we started the first inbreeding generation with a B‐S cross, reflecting the actual experimental regime. Diploid females and haploid males were randomly selected from among the offspring to carry out the inbreeding crosses. We assumed that all CSD loci segregated independently, and that diploid males were fully viable. For both species and for each number of CSD loci considered, we carried out 10 000 replicates in which we recorded the simulated operational sex ratios (haploid plus diploid individuals) as well as the diploid sex ratio (proportion of males among diploid individuals).

### Antibiotic treatment and Wolbachia titer determination

Thelytokous *L. clavipes* females were treated with a series of tetracycline concentrations (Sigma‐Aldrich) to obtain females with different *Wolbachia* titers. The procedure involved antibiotic feeding of the host larvae, adapted from Dedeine *et al*. (2001) and Ma *et al*. (2015). The applied concentrations of antibiotics were: 1.000, 0.500, 0.250, 0.125, 0.063, 0.031 and 0.016 mg/g (tetracycline/yeast). A subset of females from the first filial generation was tested for *Wolbachia* titer. Other females were mated and produced the next generation for offspring sex and ploidy determination. In a follow‐up experiment, an antibiotic concentration of 0.375, 0.281, 0.211, 0.158, 0.119, 0.089 to 0.067 mg/g (tetracycline/yeast) was applied to increase resolving power of the treatment.

*Wolbachia* titer in treated and control females was determined by quantitative real‐time PCR using the ratio of the *Wolbachia*‐specific gene Glutamate synthase subunit beta (*gssb*) and the *L. clavipes‐*specific gene Elongation factor 1‐alpha (*Lc‐ef1a*). Whole‐body DNA of individual females was isolated following a high‐salt protocol (adapted from Aljanabi & Martinez 1997). Quantitative (q)PCR was performed on an Applied Biosystems 7300 Real‐Time PCR System. Each 20‐*μ*l qPCR reaction consisted of 5 *μ*L of 10 fold cDNA dilution, 250 nmol/L forward primer and reverse primers each (gssb_wLcla_F1/R1: 5′‐GATGATGTGCTTAGTTTACCGT‐3′/5′‐CATACTCTGCTACTCCACCA‐3′; ef1a_Lcla_dna_F1/R1: 5′‐TTCCGAGTCTGTTGATTGCC‐3′/5′‐CCTGTAATAGTCTGCCTTCCC‐3′), and 1× PerfeCTa SYBR Green FastMix (QIAGEN Beverly). The qPCR program consisted of  min of denaturing at 95 °C, followed by 45 cycles of denaturing at 95 °C for 15 s, annealing at 57 °C for both *gssb* and *Lc‐Ef1a* for 30 s, extending at 72 °C for 30 s, and a final dissociation step at 95 °C for 15 s, 60 °C for 1 min, 95 °C for 15 s and 60 °C for 15 s. The relative *Wolbachia* titer was expressed as the ratio of 2 fold *gssb* DNA to *Lc‐ef1a* DNA, as a single cell of a diploid female contains two copies of *Lc‐ef1a*.

### Progeny sex and ploidy screening

The number of male and female offspring produced by females after antibiotic treatment were counted to determine the effect of *Wolbachia* titer on the progeny sex ratio. The number of hatched flies and non‐emerged fly host pupae were counted to assess wasp survival rates, and to account for potential diploid males that died during development. The ploidy level of male offspring was determined by flow cytometry as an additional test for the production of diploid males.

### Statistical analysis

All statistical analyses were performed in R (v. 3.5.1 R core team, 2019). For the inbreeding experiment, sex ratio, adult wasp proportion and non‐emerged fly pupa proportion were analyzed with generalized linear mixed effects models (GLMMs, package “lme4”, Bates *et al*. [2014] with binomial errors). Within each experiment, GLMMs were fitted using family as a random effect variable and generation (outcross, M‐S, B‐S 1–6), treatment (experimental and control groups), and their interaction as the fixed effect variables. All models were simplified from full models that included all parameters by excluding the non‐significant parameters (Crawley, 2015). Brood size data of *L. heterotoma* and *L. clavipes* were analyzed with GLMMs with a Poisson error distribution, including the interaction between generations and treatments as the fixed effect variable, and family within each experiment as a random effect variable. Pairwise comparisons of sex ratio, adult wasp proportion, non‐emerged fly pupa proportion and brood size data were performed by Tukey contrasts with Bonferroni correction using R package estimated marginal means (emmeans) (Lenth, 2020). For *Wolbachia* titer determination, a Kruskal–Wallis test was used to compare females under different tetracycline treatments and differences were subsequently analyzed using Dunn's test for multiple comparisons (Ogle *et al*., 2019).

## Results

### All‐male broods

We start by considering the potential confounding variables during the experiment that needed to be controlled for. Some females may have remained unmated and hence produced all‐male (haploid) offspring. Pooling over all generations and experiments for *L. heterotoma*, we found 45% (350 out of 781) all‐male broods in the inbreeding group compared to 61% (343 out of 559) in the control group (binomial test, *P < *0.0001). For *L. clavipes* we observed 72% (762 out of 1054) all‐male broods in the inbred group and 90% (704 out of 783) all‐male broods in the control group (binomial test, *P < *0.0001). The reason for this higher proportion of all‐male broods among controls is unknown, but it was not a strain‐specific effect, as it was observed for both reciprocal crosses (Table S1 and S4). As the inbreeding groups had lower proportions of all‐male progenies than the control groups, and these proportions did not increase over successive generations, as would be predicted under CSD, all‐male broods were considered the result of unmated females and excluded from the data analysis.

### Progeny sex ratios

**Fig. 1:**
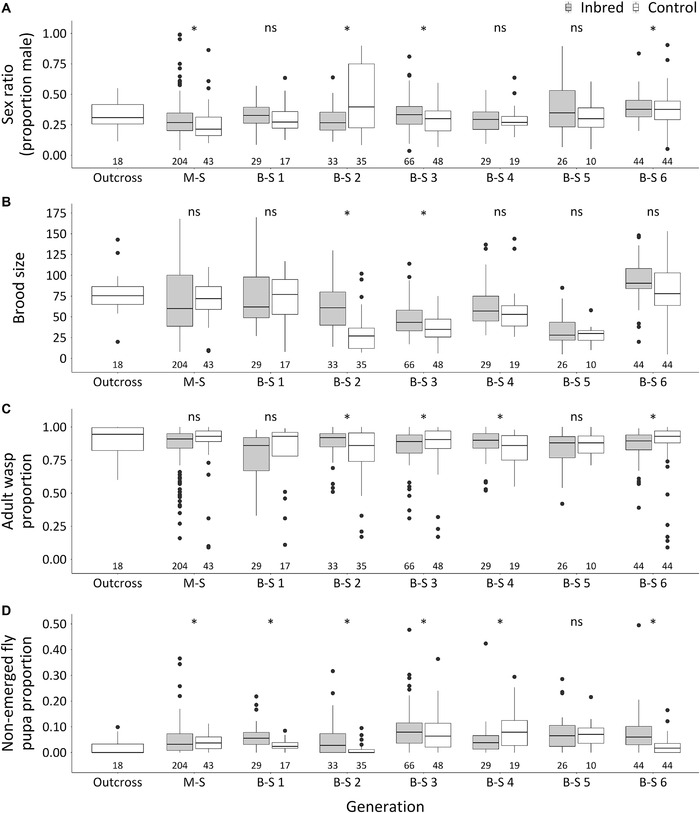
Sex ratio (A), brood size (B), adult wasp proportion (C) and non‐emerged fly pupa proportion (D) of initial outcross and successive generations of inbred and control groups in *Leptopilina heterotoma*. M‐S and B‐S indicate mother–son cross and brother–sister cross, respectively. Central box of the plot indicates the interquartile range and the internal line represents the median. The whiskers indicate 1.5 fold the interquartile range. Black dots represent extremes. Significant differences between estimated marginal means (EMMs) of inbred and control groups within generations are indicated by asterisks (*Tukey test *P* < 0.05, ns = not significant). The numbers below the plots are the sample sizes of each group and generation.

For *L. heterotoma*, M‐S crosses yielded an average progeny sex ratio of 0.29 ± 0.01 SE which was not significantly deviating from the first outcross generation (Fig. 1A). Progeny sex ratios of subsequent B‐S crosses varied from 0.33 ± 0.02 to 0.40 ± 0.02 SE and did not increase over 4–6 successive generations (Fig. 1A). Moreover, progeny sex ratios of inbred generations did not deviate systematically from their corresponding controls. These results indicate absence of CSD in *L. heterotoma*.

**Fig. 2:**
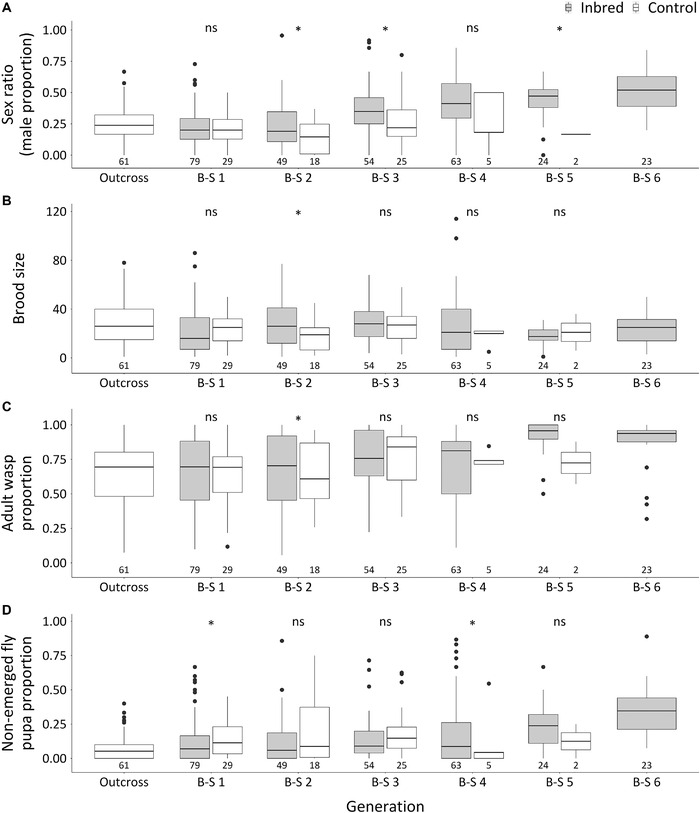
Sex ratio (A), brood size (B), adult wasp proportion (C) and non‐emerged fly pupae proportion (D) of initial outcross and successive generations of inbred and control groups in *Leptopilina clavipes*. B‐S indicates brother–sister cross. Central box of the plot indicates the interquartile range and the internal line represents the median. The whiskers indicate 1.5 fold the interquartile range. Black dots represent extremes. Significant differences between estimated marginal means (EMMs) of inbred and control groups within generations are indicated by asterisks (*Tukey test *P < *0.05, ns = not significant). The numbers below the plots are the sample sizes of each group and generation.

In *L. clavipes*, progeny sex ratios of inbred generations were higher than the corresponding control groups, except for B‐S 1 and 4 (Fig. 2A). Sex ratios also increased over successive inbreeding generations, starting from 0.36 ± 0.03 SE at B‐S 3 up to 0.51 ± 0.04 SE at B‐S 6 (Fig. 2A; Table S5). Such an increase in sex ratio is indicative of CSD but other data (below) do not support this.

### Diploid males

In *L. heterotoma*, no diploid males were detected among 80 randomly selected males from the B‐S 3 generation. To test for potential inviable diploid males, we compared brood sizes (proportion adult wasps) and proportions of non‐emerged flies (the hosts) over successive generations. There was no decrease in brood size among generations, and brood size of B‐S 6 was higher, rather than lower, than the other inbred generations (Table S2). This higher brood size of B‐S 6 is most likely due to a higher host quality as the brood size of the corresponding control was the highest of all generations as well (Table S2). If diploid males were produced but died during development, this would be reflected in an increase of progeny sex ratio, a reduction of brood size, a decrease in adult wasp proportion as well as an increase in non‐emerged fly pupae. In B‐S 2 and 3, brood size of the inbred group was higher than the control group (*P* < 0.05, Tukey pairwise comparison by Bonferroni correction), opposite to what is predicted under diploid male inviability (Fig. 1B). Adult wasp proportions varied between 0.78 ± 0.03 to 0.87 ± 0.02 in the inbreeding group and was not consistently lower than controls, neither did it decrease over successive generations (Fig. 1C, Table S3). Similarly, the proportions of non‐emerged fly pupae did not differ consistently between experimental and control groups and did not increase over inbreeding generations (Fig. 1D, Table S3). We conclude that there are no indications for inviable diploid male production resulting from inbreeding, which is consistent with an absence of CSD in *L. heterotoma*.

For *L. clavipes*, despite an increase in progeny sex ratios over generations, no adult diploid males were found among 109 tested males in the last two inbred generations (B‐S 5 and B‐S 6). These results indicate that the increase in male production in the course of inbreeding has another cause than production of diploid males. Although sample sizes decreased in the course of the experiment, brood sizes of B‐S 1 to B‐S 6 did not differ significantly (Fig. 2B; Table S6) and adult wasp proportion did not systematically decrease from B‐S 1 to B‐S 4, but increased to 0.91 ± 0.03 in B‐S 5 (Fig. 2C; Table S6). Although these data suggest that the parasitization rate of *L. clavipes* increased in the inbred group, adult wasp proportions of inbred groups were not consistently different from controls (Fig. 2C; Table S6). Non‐emerged fly pupa proportions increased over inbred generations (Fig. 2D), which could indicate the production of inviable diploid males. In B‐S 4 to 6, non‐emerged pupae were opened to determine their sex. In total, 520 out of 605 dead wasps (86%) were females among 43 out of 110 broods tested. Therefore, the increased non‐emerged fly pupa proportion was also unlikely due to diploid male production. The increased sex ratio over successive generations is most likely due to decreased viability of female wasps, indicating an effect of inbreeding depression. This may also explain why the proportion of unmated females increased, resulting in a reduction of sample size over the course of the experiment. We conclude that the inbreeding procedure indicates absence of CSD in *L. clavipes*.

### Simulations

**Fig. 3:**
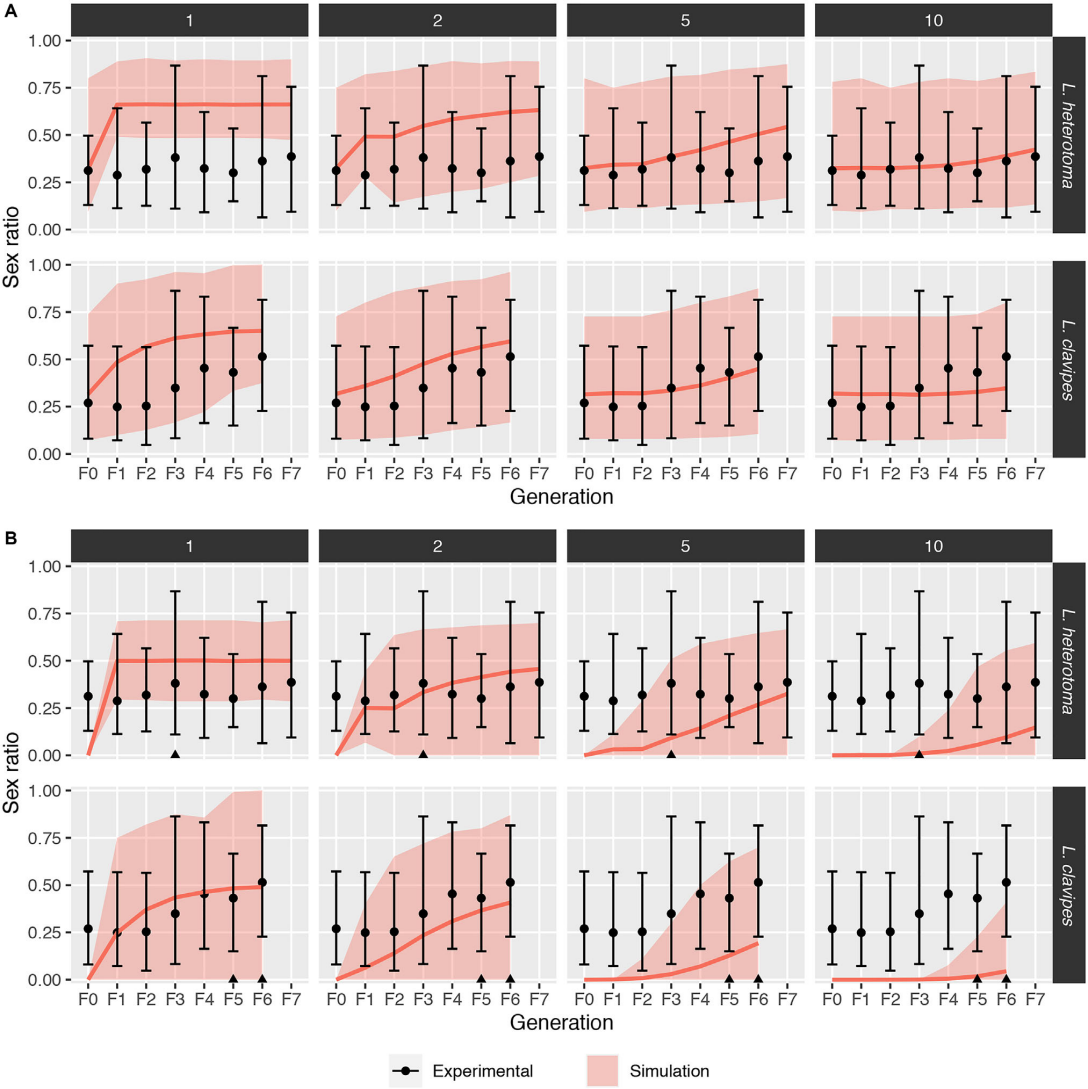
Simulated sex ratios under complementary sex determination (CSD) versus observed sex ratios. (A) Operational sex ratio (total males/total offspring). (B) Diploid sex ratio (diploid males / [diploid males + females]). In (A) and (B), the experimental sex ratio refers to the proportion of male offspring observed in the inbreeding experiment; note that for B, this does not equal the observed diploid sex ratio as we did not detect any diploid males (indicated by black triangles). Points indicate mean offspring sex ratio whereas whiskers indicate the 95% confidence interval (CI). Shaded areas indicate the 95% CI for simulated sex ratios (A = OSR, B = diploid SR) assuming CSD (*n* = 10 000 replicates); solid pink lines indicate the mean simulated sex ratio. Horizontal strips indicating the number of unlinked CSD loci, whereas vertical strips indicate species. All F0 crosses are outcrosses; F1 crosses are either mother–son (*Leptopilina heterotoma*) or brother‐sister crosses (*Leptopilina clavipes*); all subsequent crosses are brother–sister crosses for both species.

Simulations were used to predict proportions of diploid males (proportion male in diploids) and progeny sex ratio (proportion male) under 1, 2, 5 and 10 CSD loci based on our actual data. In *L. heterotoma*, following an M‐S cross, 50% of all diploid (fertilized) eggs were expected to be homozygous in the next generation under sl‐CSD, whereas this percentage would be lower with ml‐CSD, but approaching 50% over successive inbreeding generations (Fig. 3). For both *L. heterotoma* and *L. clavipes*, we found that the observed sex ratios did not deviate from the expected operational sex ratio under both sl‐ and ml‐CSD (Fig. 3A). This lack of power is different from the simulation results of, for example, Ma *et al*. (2013), which was caused by low brood sizes in our experiments. However, the observed absence of diploid males in our experiment differed markedly from the expected diploid sex ratio under sl‐CSD in *L. heterotoma* (Fig. 3B, top left panel), where a diploid sex ratio of approximately 0.3 to 0.7 is expected. In all other simulations, the absence of diploid males did not deviate as strongly from the simulated diploid sex ratios, though the overall absence of diploid males in the inbreeding experiments may nonetheless be sufficient proof for absence of CSD in both species (see Discussion).

### Wolbachia titer of antibiotic‐treated thelytokous L. clavipes females

To examine whether *Wolbachia* actively feminizes diploid embryos to induce thelytoky, a series of *Wolbachia* densities in thelytokous females was generated by antibiotic treatments. *Wolbachia* titer decreased in thelytokous females treated with an increasing antibiotic concentration (Kruskal–Wallis test, *χ* ^2^ = 43.6, *P* < 0.0001; Fig. S3A). *Wolbachia* titers of females treated with 0.016–0.125 mg/g varied between 6.15 ± 0.27 and 5.47 ± 0.38 SE, and were lower than those of control females (6.10 ± 0.32 and 6.44 ± 0.38 SE for water and ethanol control respectively), albeit not significantly (Fig. S3A). Females treated with antibiotic concentrations of 0.250 mg/g and higher had a significantly lower *Wolbachia* titer than controls (Dunn's tests, *P* < 0.05). Females treated with a concentration of 1 mg/g had the lowest *Wolbachia* titer of 0.02 ± 0.01, indicating almost a complete removal of *Wolbachia*. The increasing antibiotic concentrations yielded a stepwise decrease of *Wolbachia* titer.

### Occurrence and ploidy of male L. clavipes offspring after antibiotic treatment

Offspring sex of thelytokous females changed from only females to only males when *Wolbachia* titer was decreased (Fig. S3B). Control females produced only daughters (*n* = 12 and 10 progenies for control females treated with water and ethanol, respectively). Females treated with concentrations of 0.004–0.125 mg/g tetracycline also produced only daughters, in line with the observation that *Wolbachia* titers of these females were not significantly different from those of the control females. At concentrations of 0.25 mg/g or higher, treated females had a significantly lower *Wolbachia* titer than controls, and overall 65% males were produced among seven progenies. At an antibiotic concentration of 0.5 and 1 mg/g, only male offspring were produced. To increase the resolution within the critical antibiotic concentration range, where the production of females shifts to males, in a second experiment females were treated with 0.067–0.281 mg/g tetracycline/yeast. These females produced 3%–97% of daughters, whereas females treated with 0.375 mg/g produced only sons. This indicates that a critical *Wolbachia* titer is required for feminization of the offspring.

All males from mixed broods were haploid (98 out of 236 total), including 39 out of 99 from the first dilution series and 59 out of 137 from the second dilution series. All females from these mixed broods were diploid (35 tested out of 118; 13 out of 54 and 22 out of 64 from the first and second dilution series, respectively). Males from all‐male broods from treatments with higher antibiotic concentrations were all haploid (59 tested out of 130). Thus, no diploid males were observed among offspring of antibiotic treated *L. clavipes* females, not even in the cases of intermediate *Wolbachia* titers. If diploid males were produced but died during development, this would have led to a reduction of offspring number and an increase in the number of emerged or non‐emerged flies. The number of offspring varied with different antibiotic concentrations (Kruskal–Wallis test, *χ* ^2^ = 28.346, *P* = 0.041) but were generally not lower than that of control females and even higher from concentrations 0.125 mg/g onwards females (Table 2). The number of emerged flies and non‐emerged flies did not increase after antibiotic treatment (Kruskal–Wallis test, flies: *χ* ^2^ = 15.4, *P* = 0.565; non‐emerged flies: *χ* ^2^ = 18.6, *P* = 0.349; Table 2). Therefore, there were no indications of reduced wasp viability. Together, the antibiotic treatment results demonstrate that *Wolbachia* either only diploidizes unfertilized eggs which leads to female development or both diploidizes and actively feminizes unfertilized eggs of *L. clavipes*.

**Table 2:** Progenies of thelytokous females after antibiotic treatment

Experimental group	Antibiotic concentration (mg tetracycline/g yeast)	No. parasitizing females	No. progenies	No. (proportion) male offspring	No. males (No. diploid) tested for ploidy level	No. emerged flies	No. non‐emerged flies
Control	0 (Water)	1	12	0 (0)	0 (0)	8	1
	0 (Ethanol)	4	10	0 (0)	0 (0)	90	3
Treatment 1	0.004	3	42	0 (0)	0 (0)	56	5
	0.008	5	33	0 (0)	0 (0)	108	1
	0.016	2	2	0 (0)	0 (0)	36	0
	0.031	5	12	0 (0)	0 (0)	116	3
	0.063	1	2	0 (0)	0 (0)	21	0
	0.125	6	38	0 (0)	0 (0)	89	6
	0.25	7	153	99 (65)	39 (0)	107	9
	0.5	6	36	36 (100)	17 (0)	83	4
	1	8	62	62 (100)	25 (0)	108	3
Treatment 2	0.067	3	19	10 (53)	9 (0)	52	3
	0.089	5	37	1 (3)	1 (0)	75	1
	0.119	2	5	4 (80)	4 (0)	22	1
	0.158	5	14	6 (43)	3 (0)	90	4
	0.211	5	59	51 (86)	12 (0)	87	2
	0.281	8	67	65 (97)	30 (0)	131	2
	0.375	4	32	32 (100)	16 (0)	95	2

## Discussion

Our results conclusively reject the presence of CSD in *L. heterotoma* and *L. clavipes*. We attempted to control for a number of potentially confounding factors. First, unmated females produce only male offspring, which will inflate progeny sex ratios. Although high numbers of all‐male broods were found for both species, these were not higher in the inbred than control groups and did not increase over successive generations of inbreeding. We interpreted them as being most likely the result of non‐mating, which we frequently observe in our regular cultures, and all‐male brood producing females were therefore excluded from our analyses. For *L. heterotoma*, there were no other indications for (inviable) diploid male production following inbreeding, as we found no increase in progeny sex ratios (proportion males), no decrease of brood size, no decrease of adult wasp numbers and no increase of non‐emerged fly pupae. These results are consistent with the observation that diploid males, if produced at all, are viable. Only one out of 24 studies reported complete inviability of diploid males in Hymenoptera (Harpur *et al*., 2013).

Hey and Gargiulo (1985) reported that six generations of inbreeding in *L. heterotoma* did not increase progeny sex ratio compared to outbred control populations, but they did not determine the sex ratio for every generation. They found an average sex ratio of approximately 0.65 in their cultures, compared to 0.32 in our study. This may be caused by the fact all‐male broods were not excluded from their analysis, but they do not provide this information. If we include all in our analysis, overall sex ratios would increase to 0.55 for *L. heterotoma*. Thus, our results are comparable with those of Hey and Gargiulo (1985), and not only provide additional evidence for the absence of sl‐CSD but also refute ml‐CSD in *L. heterotoma*.

For *L. clavipes*, the results are a bit more complex. Progeny sex ratios increased in the course of inbreeding, but simultaneously the proportion of non‐emerged fly pupa increased. This could be indicative for an increasing production of inviable diploid males, but the non‐emerged wasps contained in the fly pupae were mostly females. Hence, the increase in progeny sex ratios appears to be due to a decrease in diploid female survival. This points at an inbreeding depression effect, namely reduced survival of diploid females following an increase in homozygosity, which is to be expected to be more prominent in females of haplodiploid species, as they are the diploid sex (Werren, 1993; Henter, 2003). In addition, no diploid males were detected by flow cytometry in generations 5 and 6 of inbreeding.

As an additional support for our conclusion of CSD absence, we performed simulations of our data to predict diploid male production and progeny sex ratios under CSD with 1, 2, 5 and 10 loci. In the simulations, we found that only under sl‐CSD in *L. heterotoma* the 95% confidence intervals (CIs) for the diploid sex ratio did not include 0% males. Although the 95% CI for the simulated diploid sex ratios for all other categories include the observed diploid sex ratio of 0%, these results do not necessarily support the presence of ml‐CSD in *L. heterotoma* or sl‐ or ml‐CSD in *L. clavipes*. This is because these CIs refer to the progeny sex ratios of individual broods, rather than the sex ratio taken across all broods. Under these scenarios, the probability of observing no diploid males in a single brood may be non‐zero, but the probability of observing no diploid males in all broods decreases nonetheless. Taken across all investigated broods, we may therefore conclude that the lack of diploid males lends strong support for absence of CSD in both species.

This is only the third study, following Biémont and Bouletreau (1980) and Hey and Gargiulo (1985), to test and report absence of CSD in species of the family Figitidae within the superfamily Cynipoidea. The absence of CSD in *L. heterotoma* and *L. clavipes* is consistent with the prediction of Asplen *et al*. (2009) that CSD is absent in the superfamily Cynipoidea, in which endosymbiont‐induced thelytokous reproduction is widespread. Indeed, thelytoky in *L. clavipes* is caused by gamete duplication (Pannebakker *et al*., 2004b), which is the most commonly observed mechanism of cytologically investigated cases of *Wolbachia*‐induced thelytoky in Hymenoptera (Ma & Schwander, 2017). However, *Wolbachia‐*induced thelytoky is also known to occur in other than the hymenopteran superfamilies Chalcidoidea and Cynipoidea. An example is *A. japonica*, that belongs to the family of Braconidae within the superfamily Ichneumonoidea (Kremer *et al*., 2009). Beukeboom *et al*. (2000) and Ma *et al*. (2013) reported absence of CSD in *A. japonica* and three other *Asobara* species. This is consistent with the conclusion of Asplen *et* *al*. (2009) that multiple switches to ml‐CSD and non‐CSD have occurred in the Braconidae and that taxonomic classification alone is not sufficient to predict presence or absence of CSD. Moreover, our current results indicate that CSD may not necessarily be mechanistically incompatible with endosymbiont‐induced thelytoky involving gamete duplication. Ma *et al*. (2015) reported a two‐step mechanism for *Wolbachia‐*induced thelytoky in *A. japonica*, consisting of diploidization and feminization of unfertilized eggs, whereby the two steps require a different *Wolbachia* titer. We also experimentally varied *Wolbachia* titer in *L. clavipes* but did not find evidence for separate diploidization and feminization. These results can be explained in two ways. First, *Wolbachia* may not actively feminize diploidized unfertilized eggs, implicating that diploidy alone is sufficient for female development, possibly through a dose‐dependent effect on host sex determination. This would in any case exclude a CSD mechanism. Alternatively, a two‐step process could also apply for *L. clavipes*, but in this case the two steps do not differ in *Wolbachia* titer requirements. Under this scenario, female development under thelytoky in *L. clavipes*, similar to *A. japonica*, does not result from diploidy alone and needs an additional activating step, performed by the endosymbiont. Therefore, CSD and endosymbiont‐induced thelytoky involving gamete duplication may be compatible in theory, if the endosymbiont can provide a substitute for the CSD feminization mechanism. Although this has until now never been reported to simultaneously occur in a single species, we recommend to consider this option in future studies.

Although the precise mechanisms by which *Wolbachia* induce female development from unfertilized diploid eggs is unknown, it is clearly intertwined with the sex determination system of its host. Many Hymenoptera have evolved a sex determination system, in which the primary signal is not dependent on the allelic state of a CSD locus. The sex‐determining system of *Nasonia vitripennis* is illustrative. The feminizing signal is provided by its wasp overruler of masculinisation (*wom*) gene which is active on the paternal genome but silenced on the maternal genome (Beukeboom *et al*., 2007; Verhulst *et al*., 2010; van de Zande & Verhulst, 2014; Zou *et al*., 2020). We have preliminary evidence that both in *A. japonica* and *L. clavipes*, the paternal genome provides a feminizing signal and that *Wolbachia* may be able to substitute for this paternal contribution (Geuverink, 2017) to an unfertilized diploid egg. This could imply that not the homozygotic state, but the inability of the endosymbiont to substitute for the *tra* activating mechanism of CSD is the reason for the incompatibility with endosymbiont‐induced thelytoky. Driven by the sterile diploid male production under the ancestral sl‐CSD (Naito & Suzuki, 1991; Yamauchi *et al*., 2001), other systems than CSD may have evolved that were prone to endosymbiont manipulation of the host sex determination. These issues can obviously not yet be resolved and require more detailed investigation of the molecular signals and cytogenetic mechanisms of thelytoky induction, as well as of gene regulation in haplodiploid sex‐determination pathways.

## Disclosure

The authors declare that there is no conflict of interest.

## Supporting information


**Fig. S1** Experimental set‐up of inbred and control groups in *L. heterotoma*, displaying one direction of the reciprocal cross.
**Fig. S2** Experimental set‐up of inbred and control groups in *L. clavipes*, displaying one direction of the reciprocal cross.
**Fig. S3** (A) *Wolbachia* titer as function of various tetracycline concentrations. 0 (Water) and 0 (Ethanol) are control treatments with no tetracycline. Asterisks indicate significant differences (*P < 0.05, **P < 0.01, ***P < 0.001, ****P < 0.0001) of experimental groups with 0 (Water) and hash tags (^#^ *P* < 0.05, ^##^ *P* < 0.01, ^###^ *P* < 0.001, ^####^ *P* < 0.0001) with 0 (Ethanol) control. (B) Sex and ploidy of offspring in experiment 1 and 2. X axis indicates the range of antibiotic concentrations (tetracycline/yeast, mg/g).
**Table S1** Number of hosted females, parasitizing females, females that produced daughters, females that only produced sons and females that produced no offspring in inbred and control groups over successive generations of inbreeding in *L. heterotoma*.
**Table S2** Proportion males, fertilization rate, brood size, adult wasp proportion, dead pupa proportion, flies number, dead fly pupa number and host number of females that produced daughters in the initial outcross and in inbred and control groups over successive generations of inbreeding in *L. heterotoma*.
**Table S3** Pairwise comparison of sex ratio (proportion male), brood size, adult wasp proportion and non‐emerged fly pupa proportion between outcross and inbred generations of *L. heterotoma* after GLMMs.
**Table S4** Number of hosted females, parasitizing females, females that produced daughters, females that only produced sons and females that produced no offspring in inbred and control groups over successive generations of inbreeding *L. clavipes*.
**Table S5** Sex ratio (proportion males), fertilization rate, brood size, adult wasp proportion, dead pupa proportion, flies number, dead fly pupa number and host number of females that produced daughters in the initial outcross and in inbred and control groups over successive generations of inbreeding in *L. clavipes*.
**Table S6** Pairwise comparison of sex ratio (proportion male), adult wasp proportion and non‐emerged fly pupae proportion between outcross and inbred generations of *L. clavipes* after GLMMs.

Supplementary Material
