# Supplementary material for: Absence of complementary sex determination in two Leptopilina species (Figitidae, Hymenoptera) and a reconsideration of its incompatibility with endosymbiont‐induced thelytoky
Source: Insect Sci. 2021 Oct 25;29(3):900–14. doi: 10.1111/1744-7917.12969 (PMC9297927; doi:10.1111/1744-7917.12969)
Supplement: Supplementary file 1 — Fig. S1 Experimental set‐up of inbred and control groups in L. heterotoma, displaying one direction of the reciprocal cross. Fig. S2 Experimental set‐up of inbred and control groups in L. clavipes, displaying one direction of the reciprocal cross. Fig. S3 (A) Wolbachia titer as function of various tetracycline concentrations. 0 (Water) and 0 (Ethanol) are control treatments with no tetracycline. Asterisks indicate significant differences (*P < 0.05, **P < 0.01, ***P < 0.001, ****P < 0.0001) of experimental groups with 0 (Water) and hash tags (# P < 0.05, ## P < 0.01, ### P < 0.001, #### P < 0.0001) with 0 (Ethanol) control. (B) Sex and ploidy of offspring in experiment 1 and 2. X axis indicates the range of antibiotic concentrations (tetracycline/yeast, mg/g). Table S1 Number of hosted females, parasitizing females, females that produced daughters, females that only produced sons and females that produced no offspring in inbred and control groups over successive generations of inbreeding in L. heterotoma. Table S2 Proportion males, fertilization rate, brood size, adult wasp proportion, dead pupa proportion, flies number, dead fly pupa number and host number of females that produced daughters in the initial outcross and in inbred and control groups over successive generations of inbreeding in L. heterotoma. Table S3 Pairwise comparison of sex ratio (proportion male), brood size, adult wasp proportion and non‐emerged fly pupa proportion between outcross and inbred generations of L. heterotoma after GLMMs. Table S4 Number of hosted females, parasitizing females, females that produced daughters, females that only produced sons and females that produced no offspring in inbred and control groups over successive generations of inbreeding L. clavipes. Table S5 Sex ratio (proportion males), fertilization rate, brood size, adult wasp proportion, dead pupa proportion, flies number, dead fly pupa number and host number of females that produced daughters in the initial outcross [file INS-29-900-s001.docx]

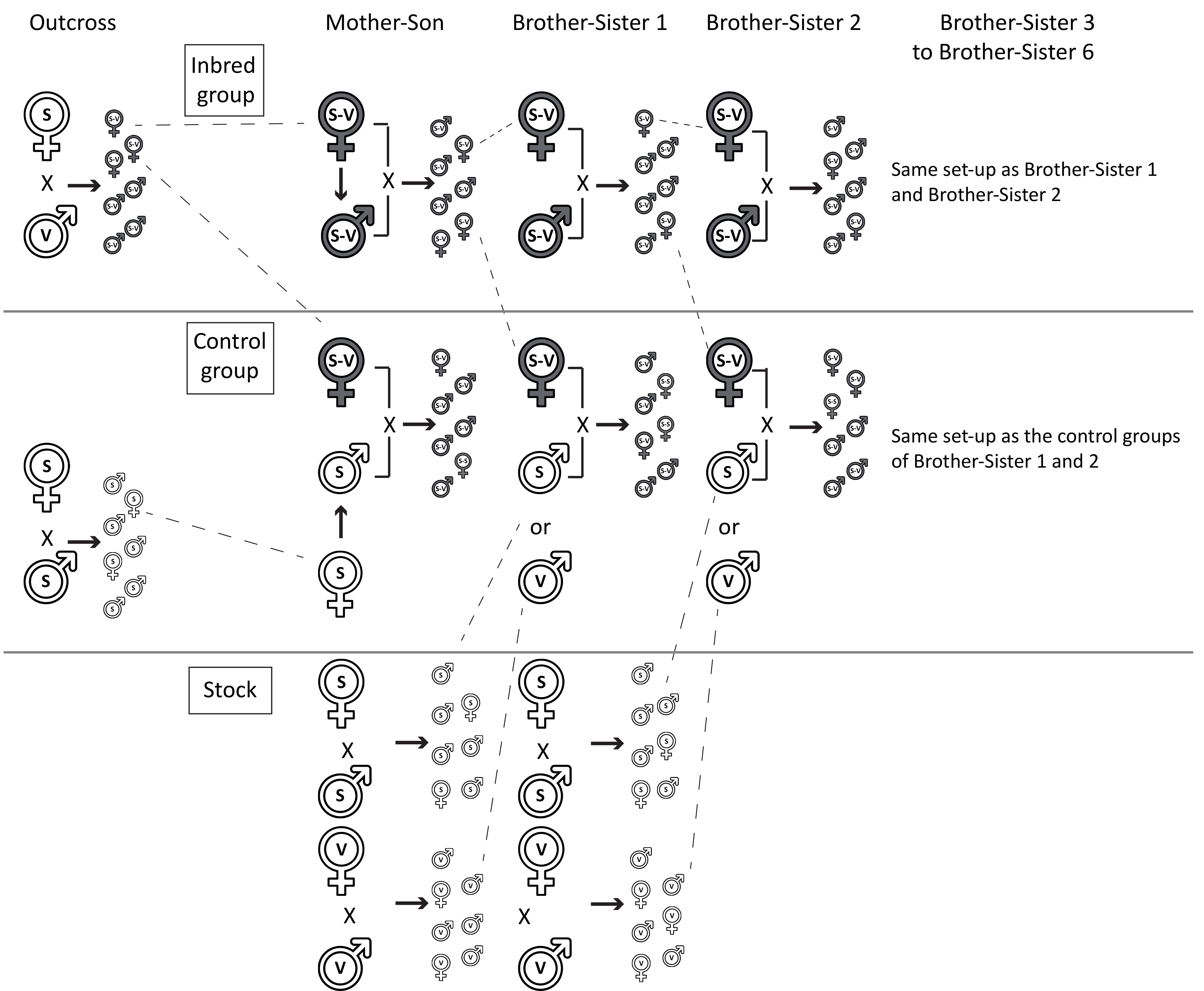


**Figure S1**. Experimental set-up of inbred and control groups in *L. heterotoma*, displaying one direction of the reciprocal cross. A Mother-Son cross is followed by 6 generations of Brother-Sister crosses. Character S and V represent strain SC and VB, S-V represents the strain generated by the outcross between SC female and VB male.


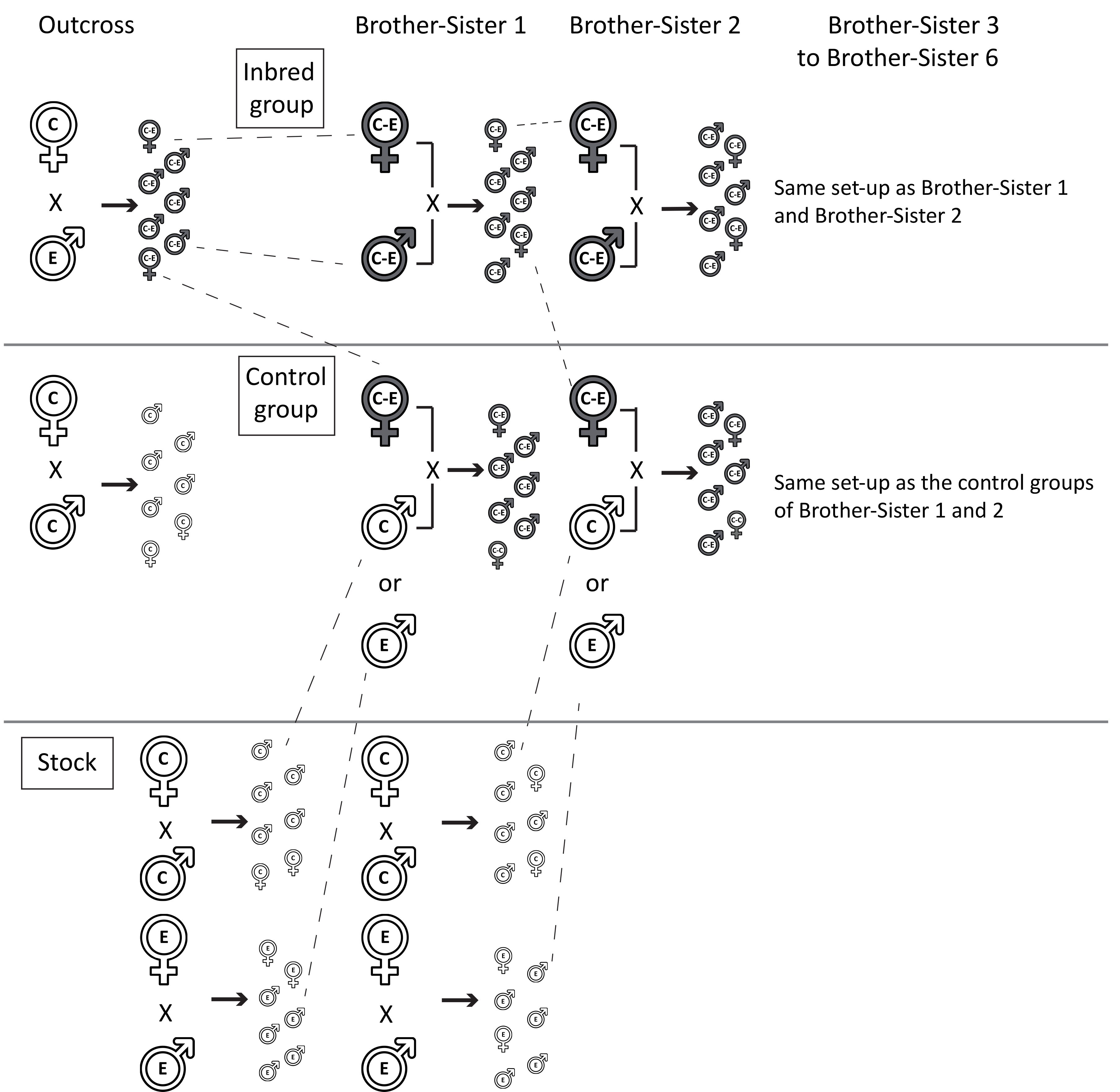


**Figure S2**. Experimental set-up of inbred and control groups in *L. clavipes*, displaying one direction of the reciprocal cross. As a Mother-Son cross was not possible there are 6 generations of Brother-Sister crosses. Character C and E represent strain CA1 and EPG2, C-E represents the strain generated by the outcross between CA1 female and EPG2 male.

**Table S1**. Number of hosted females, parasitizing females, females that produced daughters, females that only produced sons, and females that produced no offspring in inbred and control groups over successive generations of inbreeding in *L. heterotoma*. SC-VB represents the strain generated by the outcross between SC female and VB male, VB-SC represents the strain generated by the outcross between VB female and SC male. The bold numbers indicate significant higher proportions in the inbred group than the corresponding control group.

|  |  | Inbred group | | | Control group | | |
| --- | --- | --- | --- | --- | --- | --- | --- |
|  | Generation | No. hosted females | No.  (percentage) parasitizing females | No.  (percentage) females that only produced sons | No. hosted females | No.  (percentage) parasitizing females | No.  (percentage) females that only produced sons |
| Overall | M-S | 353 | 299 (85) | 95 (32) | 94 | 80 (85) | 37 (46) |
|  | B-S 1 | 147 | 126 (86) | 97 (77) | 148 | 132 (89) | 115 (87) |
|  | B-S 2 | 120 | **113 (94)** | **80 (71)** | 119 | 103 (87) | 68 (66) |
|  | B-S 3 | 106 | 91 (86) | 25 (27) | 104 | 92 (88) | 44 (48) |
|  | B-S 4 | 55 | 52 (95) | 23 (44) | 56 | 54 (96) | 35 (65) |
|  | B-S 5 | 50 | **48 (96)** | 22 (46) | 48 | 45 (94) | 35 (78) |
|  | B-S 6 | 54 | 52 (96) | 8 (15) | 53 | 53 (100) | 9 (17) |
|  | Total | 885 | 781 (88) | 350 (45) | 622 | 559 (90) | 343 (61) |
| SC-VB | M-S | 199 | 166 (83) | 45 (27) | 50 | 46 (92) | 18 (39) |
|  | B-S 1 | 79 | 68 (86) | 55 (81) | 78 | 73 (94) | 63 (86) |
|  | B-S 2 | 57 | **52 (91)** | **44 (85)** | 56 | 49 (87) | 34 (69) |
|  | B-S 3 | 46 | **43 (93)** | 13 (30) | 45 | 41 (91) | 16 (39) |
|  | B-S 4 | 16 | **15 (94)** | **9 (60)** | 15 | 14 (93) | 8 (57) |
|  | B-S 5 | 11 | **11 (100)** | 3 (27) | 10 | 9 (90) | 6 (67) |
|  | B-S 6 | 14 | 14 (100) | 4 (29) | 14 | 14 (100) | 4 (29) |
|  | Total | 422 | 369 (87) | 173 (47) | 268 | 266 (99) | 149 (56) |
| VB-SC | M-S | 154 | **133 (86)** | 50 (38) | 44 | 34 (77) | 19 (56) |
|  | B-S 1 | 68 | **58 (85)** | 42 (72) | 70 | 59 (84) | 52 (88) |
|  | B-S 2 | 63 | **61 (97)** | 36 (59) | 63 | 54 (86) | 34 (63) |
|  | B-S 3 | 60 | 48 (80) | 12 (25) | 59 | 51 (86) | 28 (55) |
|  | B-S 4 | 39 | 37 (95) | 14 (38) | 41 | 40 (98) | 27 (68) |
|  | B-S 5 | 39 | 37 (95) | 19 (51) | 38 | 36 (95) | 29 (81) |
|  | B-S 6 | 40 | 38 (95) | 4 (11) | 39 | 39 (100) | 5 (13) |
|  | Total | 463 | **412 (89)** | 177 (43) | 354 | 313 (88) | 194 (62) |

**Table S2**. Proportion males, fertilization rate, brood size, adult wasp proportion, dead pupa proportion, flies number, dead fly pupa number, and host number of females that produced daughters in the initial outcross, and in inbred and control groups over successive generations of inbreeding in *L. heterotoma*. M-S is mother-son cross, B-S = brother sister cross.

| Treatment | Generation | No. females | Proportion male (Mean ± SE) | | | Fertilization rate (Mean ± SE) | | | Brood size (Mean ± SE) | | | Adult wasp proportion (Mean ± SE) | | | Dead fly pupa proportion (Mean ± SE) | | | No. Flies (Mean ± SE) | | | No. dead fly pupa (Mean ± SE) | | | No. Host (Mean ± SE) | | |
| --- | --- | --- | --- | --- | --- | --- | --- | --- | --- | --- | --- | --- | --- | --- | --- | --- | --- | --- | --- | --- | --- | --- | --- | --- | --- | --- |
|  |  |  |  |  |  |  |  |  |  |  |  |  |  |  |  |  |  |  |  |  |  |  |  |  |  |  |
| Inbred group | Outcross | 18 | 0.33 | ± | 0.03 | 0.67 | ± | 0.03 | 78 | ± | 6 | 0.89 | ± | 0.03 | 0.02 | ± | 0.01 | 12 | ± | 5 | 3 | ± | 1 | 92 | ± | 11 |
|  | M-S | 204 | 0.29 | ± | 0.01 | 0.71 | ± | 0.01 | 69 | ± | 3 | 0.86 | ± | 0.01 | 0.05 | ± | 0 | 7 | ± | 1 | 3 | ± | 0 | 79 | ± | 3 |
|  | B-S 1 | 29 | 0.33 | ± | 0.02 | 0.67 | ± | 0.02 | 71 | ± | 6 | 0.78 | ± | 0.03 | 0.07 | ± | 0.01 | 15 | ± | 5 | 6 | ± | 1 | 92 | ± | 8 |
|  | B-S 2 | 33 | 0.29 | ± | 0.02 | 0.71 | ± | 0.02 | 63 | ± | 5 | 0.87 | ± | 0.02 | 0.06 | ± | 0.01 | 5 | ± | 1 | 4 | ± | 1 | 72 | ± | 6 |
|  | B-S 3 | 66 | 0.35 | ± | 0.02 | 0.65 | ± | 0.02 | 47 | ± | 3 | 0.84 | ± | 0.02 | 0.09 | ± | 0.01 | 4 | ± | 1 | 5 | ± | 0 | 56 | ± | 3 |
|  | B-S 4 | 29 | 0.3 | ± | 0.02 | 0.7 | ± | 0.02 | 63 | ± | 5 | 0.85 | ± | 0.03 | 0.05 | ± | 0.01 | 7 | ± | 1 | 4 | ± | 1 | 73 | ± | 5 |
|  | B-S 5 | 26 | 0.38 | ± | 0.04 | 0.62 | ± | 0.04 | 34 | ± | 4 | 0.84 | ± | 0.03 | 0.08 | ± | 0.01 | 3 | ± | 1 | 3 | ± | 1 | 39 | ± | 4 |
|  | B-S 6 | 44 | 0.39 | ± | 0.02 | 0.61 | ± | 0.02 | 94 | ± | 4 | 0.85 | ± | 0.02 | 0.08 | ± | 0.01 | 10 | ± | 2 | 9 | ± | 1 | 112 | ± | 5 |
| Control group | Outcross | 14 | 0.3 | ± | 0.02 | 0.7 | ± | 0.02 | 91 | ± | 10 | 0.81 | ± | 0.04 | 0.05 | ± | 0.01 | 21 | ± | 10 | 5 | ± | 1 | 118 | ± | 17 |
|  | M-S | 43 | 0.26 | ± | 0.02 | 0.74 | ± | 0.02 | 72 | ± | 4 | 0.87 | ± | 0.03 | 0.04 | ± | 0 | 9 | ± | 3 | 3 | ± | 0 | 84 | ± | 3 |
|  | B-S 1 | 17 | 0.3 | ± | 0.04 | 0.7 | ± | 0.04 | 75 | ± | 7 | 0.79 | ± | 0.07 | 0.03 | ± | 0.01 | 20 | ± | 7 | 3 | ± | 0 | 97 | ± | 7 |
|  | B-S 2 | 35 | 0.47 | ± | 0.05 | 0.53 | ± | 0.05 | 30 | ± | 4 | 0.79 | ± | 0.04 | 0.01 | ± | 0 | 7 | ± | 2 | 1 | ± | 0 | 38 | ± | 4 |
|  | B-S 3 | 48 | 0.29 | ± | 0.02 | 0.71 | ± | 0.02 | 36 | ± | 2 | 0.86 | ± | 0.03 | 0.08 | ± | 0.01 | 3 | ± | 1 | 3 | ± | 0 | 42 | ± | 2 |
|  | B-S 4 | 19 | 0.3 | ± | 0.03 | 0.7 | ± | 0.03 | 58 | ± | 7 | 0.83 | ± | 0.03 | 0.1 | ± | 0.02 | 6 | ± | 2 | 7 | ± | 1 | 70 | ± | 8 |
|  | B-S 5 | 10 | 0.31 | ± | 0.05 | 0.69 | ± | 0.05 | 29 | ± | 4 | 0.87 | ± | 0.03 | 0.08 | ± | 0.02 | 2 | ± | 1 | 3 | ± | 1 | 34 | ± | 5 |
|  | B-S 6 | 44 | 0.38 | ± | 0.03 | 0.62 | ± | 0.03 | 79 | ± | 5 | 0.84 | ± | 0.04 | 0.03 | ± | 0.01 | 9 | ± | 3 | 3 | ± | 1 | 91 | ± | 5 |

**Table S3**. Pairwise comparison of sex ratio (proportion male), brood size, adult wasp proportion, and non-emerged fly pupa proportion between outcross and inbred generations of *L. heterotoma* after GLMMs. Estimated marginal means (EMMs) of each generation (left column) is compared to the generation shown in the top row (* indicates significant difference, - indicates no difference based on Tukey test *P* < 0.05). Upper right half indicates the differences (± SE). M-S and B-S indicate mother-son cross and brother-sister cross.

| Sex ratio (proportion male) | | | | | |  | | | | | |  | | | | | |  | | | | |
| --- | --- | --- | --- | --- | --- | --- | --- | --- | --- | --- | --- | --- | --- | --- | --- | --- | --- | --- | --- | --- | --- | --- |
| Generation | Outcross | M-S | | | B-S 1 | | | B-S 2 | | | B-S 3 | | | B-S 4 | | | B-S 5 | | | B-S 6 | | |
| Outcross |  | -0.23 | ± | 0.07 | -0.05 | ± | 0.08 | -0.16 | ± | 0.08 | -0.51 | ± | 0.08 | -0.05 | ± | 0.08 | -0.99 | ± | 0.09 | -0.16 | ± | 0.08 |
| M-S | - |  |  |  | 0.18 | ± | 0.05 | 0.07 | ± | 0.05 | -0.28 | ± | 0.04 | 0.18 | ± | 0.05 | -0.76 | ± | 0.07 | 0.08 | ± | 0.04 |
| B-S 1 | - | * | | |  |  |  | -0.11 | ± | 0.06 | -0.45 | ± | 0.06 | 0.01 | ± | 0.07 | -0.93 | ± | 0.08 | -0.10 | ± | 0.06 |
| B-S 2 | - | - | | | - | | |  |  |  | -0.35 | ± | 0.06 | 0.11 | ± | 0.07 | -0.83 | ± | 0.07 | 0.00 | ± | 0.06 |
| B-S 3 | * | * | | | * | | | * | | |  |  |  | 0.46 | ± | 0.06 | -0.48 | ± | 0.07 | 0.35 | ± | 0.05 |
| B-S 4 | - | * | | | - | | | - | | | * | | |  |  |  | -0.94 | ± | 0.08 | -0.11 | ± | 0.06 |
| B-S 5 | * | * | | | * | | | * | | | * | | | * | | |  |  |  | 0.83 | ± | 0.07 |
| B-S 6 | - | - | | | - | | | - | | | * | | | - | | | * | | |  |  |  |
|  |  |  |  |  |  |  |  |  |  |  |  |  |  |  |  |  |  |  |  |  |  |  |
| Brood size | | | | | |  | | | | | |  | | | | | |  | | | | |
| Generation | Outcross | M-S | | | B-S 1 | | | B-S 2 | | | B-S 3 | | | B-S 4 | | | B-S 5 | | | B-S 6 | | |
| Outcross |  | 0.20 | ± | 0.12 | 0.07 | ± | 0.14 | 0.21 | ± | 0.14 | 0.51 | ± | 0.13 | 0.23 | ± | 0.15 | 0.88 | ± | 0.15 | -0.16 | ± | 0.14 |
| M-S | - |  |  |  | -0.13 | ± | 0.10 | 0.01 | ± | 0.09 | 0.31 | ± | 0.07 | 0.03 | ± | 0.10 | 0.68 | ± | 0.11 | -0.36 | ± | 0.09 |
| B-S 1 | - | - | | |  |  |  | 0.14 | ± | 0.12 | 0.44 | ± | 0.11 | 0.16 | ± | 0.13 | 0.81 | ± | 0.13 | -0.23 | ± | 0.12 |
| B-S 2 | - | - | | | - | | |  |  |  | 0.30 | ± | 0.10 | 0.02 | ± | 0.12 | 0.67 | ± | 0.13 | -0.37 | ± | 0.11 |
| B-S 3 | * | * | | | * | | | - | | |  |  |  | -0.28 | ± | 0.11 | 0.37 | ± | 0.12 | -0.67 | ± | 0.10 |
| B-S 4 | - | - | | | - | | | - | | | - | | |  |  |  | 0.65 | ± | 0.13 | -0.39 | ± | 0.11 |
| B-S 5 | * | * | | | * | | | * | | | * | | | * | | |  |  |  | -1.04 | ± | 0.12 |
| B-S 6 | - | * | | | - | | | * | | | * | | | * | | | * | | |  |  |  |
|  |  |  |  |  |  |  |  |  |  |  |  |  |  |  |  |  |  |  |  |  |  |  |
| Adult wasp proportion | | | | | |  | | | | | |  | | | | | |  | | | | |
| Generation | Outcross | M-S | | | B-S 1 | | | B-S 2 | | | B-S 3 | | | B-S 4 | | | B-S 5 | | | B-S 6 | | |
| Outcross |  | 0.07 | ± | 0.07 | -0.05 | ± | 0.08 | 0.16 | ± | 0.08 | -0.25 | ± | 0.08 | -0.17 | ± | 0.09 | 0.39 | ± | 0.10 | -0.49 | ± | 0.08 |
| M-S | - |  |  |  | -0.11 | ± | 0.05 | 0.09 | ± | 0.05 | -0.32 | ± | 0.05 | -0.24 | ± | 0.06 | 0.32 | ± | 0.07 | -0.55 | ± | 0.05 |
| B-S 1 | - | - | | |  |  |  | 0.21 | ± | 0.06 | -0.20 | ± | 0.06 | -0.12 | ± | 0.07 | 0.44 | ± | 0.08 | -0.44 | ± | 0.06 |
| B-S 2 | - | - | | | * | | |  |  |  | -0.41 | ± | 0.06 | -0.33 | ± | 0.07 | 0.23 | ± | 0.07 | -0.65 | ± | 0.06 |
| B-S 3 | - | * | | | * | | | * | | |  |  |  | 0.08 | ± | 0.07 | 0.64 | ± | 0.07 | -0.23 | ± | 0.06 |
| B-S 4 | - | * | | | - | | | * | | | - | | |  |  |  | 0.56 | ± | 0.08 | -0.32 | ± | 0.06 |
| B-S 5 | * | * | | | * | | | - | | | * | | | * | | |  |  |  | -0.88 | ± | 0.07 |
| B-S 6 | * | * | | | * | | | * | | | * | | | * | | | * | | |  |  |  |
|  | | |  |  |  |  |  |  |  |  |  |  |  |  |  |  |  |  |  |  |  |  |
| Non-emerged fly pupae proportion | | | | | |  | | | | | |  | | | | | |  | | | | |
| Generation | Outcross | M-S | | | B-S 1 | | | B-S 2 | | | B-S 3 | | | B-S 4 | | | B-S 5 | | | B-S 6 | | |
| Outcross |  | -0.89 | ± | 0.17 | -1.16 | ± | 0.18 | -0.96 | ± | 0.19 | -1.44 | ± | 0.18 | -0.94 | ± | 0.20 | -1.42 | ± | 0.21 | -1.36 | ± | 0.18 |
| M-S | * |  |  |  | -0.27 | ± | 0.09 | -0.08 | ± | 0.11 | -0.55 | ± | 0.08 | -0.05 | ± | 0.12 | -0.53 | ± | 0.13 | -0.47 | ± | 0.08 |
| B-S 1 | * | - | | |  |  |  | 0.19 | ± | 0.12 | -0.28 | ± | 0.10 | 0.22 | ± | 0.13 | -0.26 | ± | 0.15 | -0.20 | ± | 0.10 |
| B-S 2 | * | - | | | - | | |  |  |  | -0.48 | ± | 0.11 | 0.03 | ± | 0.14 | -0.46 | ± | 0.15 | -0.39 | ± | 0.11 |
| B-S 3 | * | * | | | - | | | * | | |  |  |  | 0.50 | ± | 0.12 | 0.02 | ± | 0.14 | 0.08 | ± | 0.09 |
| B-S 4 | * | - | | | - | | | - | | | * | | |  |  |  | -0.48 | ± | 0.16 | -0.42 | ± | 0.12 |
| B-S 5 | * | * | | | - | | | - | | | - | | | - | | |  |  |  | 0.06 | ± | 0.13 |
| B-S 6 | * | * | | | - | | | * | | | - | | | * | | | - | | |  |  |  |

**Table S4**. Number of hosted females, parasitizing females, females that produced daughters, females that only produced sons, and females that produced no offspring in inbred and control groups over successive generations of inbreeding *L. clavipes*. CA1-EPG2 represents the strain generated by the outcross between CA1 female and EPG2 male, EPG2-CA1 represents the strain generated by the outcross between EPG2 female and CA1 male. The bold numbers indicate significant higher proportions in the inbred group than the corresponding control group.

|  |  | Inbred group | | | Control group | | |
| --- | --- | --- | --- | --- | --- | --- | --- |
|  | Generation | No. hosted females | No. (percentage) parasitizing females | No. (percentage) females that only produced sons | No. hosted females | No. (percentage) parasitizing females | No. (percentage) females that only produced sons |
| Overall | B-S 1 | 388 | 311 (80) | 232 (70) | 260 | 221 (90) | 192 (87) |
|  | B-S 2 | 364 | 296 (81) | 247 (83) | 275 | 224 (81) | 206 (92) |
|  | B-S 3 | 255 | 195 (76) | 141 (72) | 176 | 144 (82) | 119 (83) |
|  | B-S 4 | 166 | **148 (89)** | 85 (58) | 116 | 102 (88) | 97 (87) |
|  | B-S 5 | 76 | 61 (80) | 37 (61) | 60 | 51 (85) | 49 (96) |
|  | B-S 6 | 57 | 43 (75) | 20 (47) | 47 | 41 (87) | 41 (100) |
|  | Total | 1306 | 1054 (81) | 762 (72) | 934 | 783 (84) | 704 (90) |
| CA1-EPG2 | B-S 1 | 257 | 208 (81) | 148 (67) | 157 | 130 (83) | 112 (86) |
|  | B-S 2 | 231 | 197 (85) | 183 (93) | 166 | 145 (87) | 140 (97) |
|  | B-S 3 | 107 | 77 (72) | 54 (70) | 82 | 61 (74) | 54 (89) |
|  | B-S 4 | 53 | 49 (92) | 48 (98) | 52 | 49 (94) | 49 (100) |
|  | B-S 5 | 14 | **10 (71)** | **10 (100)** | 12 | 7 (58) | 5 (71) |
|  | B-S 6 | 0 | 0 (0) | 0 (0) | 0 | 0 (0) | 0 (0) |
|  | Total | 662 | 541 (82) | 443 (82) | 469 | 392 (84) | 360 (92) |
| EPG2-CA1 | B-S 1 | 131 | 103 (79) | 84 (75) | 103 | 91 (88) | 80 (88) |
|  | B-S 2 | 133 | **99 (74)** | 64 (64) | 109 | 79 (72) | 66 (84) |
|  | B-S 3 | 148 | 118 (80) | 87 (73) | 94 | 83 (88) | 65 (78) |
|  | B-S 4 | 113 | **99 (88)** | 37 (29) | 64 | 53 (83) | 48 (91) |
|  | B-S 5 | 62 | 51 (82) | 27 (53) | 48 | 44 (92) | 44 (100) |
|  | B-S 6 | 57 | 43 (75) | 20 (47) | 47 | 41 (87) | 41 (100) |
|  | Total | 644 | 513 (80) | 319 (62) | 465 | 391 (84) | 344 (88) |

**Table S5**. Sex ratio (proportion males), fertilization rate, brood size, adult wasp proportion, dead pupa proportion, flies number, dead fly pupa number, and host number of females that produced daughters in the initial outcross, and in inbred and control groups over successive generations of inbreeding in *L. clavipes*. B-S = brother sister cross.

| Treatment | Generation | No. females | Sex ratio (roportion male,Mean ± SE) | | | Fertilization rate (Mean ± SE) | | | Brood size (Mean ± SE) | | | Adult wasp proportion (Mean ± SE) | | | Dead fly pupa proportion (Mean ± SE) | | | No. flies (Mean ± SE) | | | No. dead fly pupa  (Mean ± SE) | | | No. host (Mean ± SE) | | |
| --- | --- | --- | --- | --- | --- | --- | --- | --- | --- | --- | --- | --- | --- | --- | --- | --- | --- | --- | --- | --- | --- | --- | --- | --- | --- | --- |
| Inbred group | Outcross | 61 | 0.25 | ± | 0.02 | 0.75 | ± | 0.02 | 29 | ± | 2 | 0.64 | ± | 0.03 | 0.08 | ± | 0.01 | 21 | ± | 3 | 2 | ± | 0 | 51 | ± | 4 |
|  | B-S 1 | 79 | 0.22 | ± | 0.02 | 0.78 | ± | 0.02 | 22 | ± | 2 | 0.66 | ± | 0.03 | 0.14 | ± | 0.02 | 13 | ± | 2 | 3 | ± | 0 | 37 | ± | 2 |
|  | B-S 2 | 49 | 0.24 | ± | 0.03 | 0.76 | ± | 0.03 | 28 | ± | 3 | 0.68 | ± | 0.04 | 0.13 | ± | 0.03 | 19 | ± | 4 | 4 | ± | 1 | 51 | ± | 5 |
|  | B-S 3 | 54 | 0.36 | ± | 0.03 | 0.64 | ± | 0.03 | 29 | ± | 2 | 0.76 | ± | 0.03 | 0.15 | ± | 0.02 | 11 | ± | 2 | 5 | ± | 1 | 45 | ± | 2 |
|  | B-S 4 | 63 | 0.42 | ± | 0.03 | 0.58 | ± | 0.03 | 26 | ± | 3 | 0.7 | ± | 0.03 | 0.2 | ± | 0.03 | 10 | ± | 1 | 8 | ± | 2 | 44 | ± | 4 |
|  | B-S 5 | 24 | 0.44 | ± | 0.03 | 0.56 | ± | 0.03 | 18 | ± | 1 | 0.91 | ± | 0.03 | 0.24 | ± | 0.03 | 2 | ± | 0 | 6 | ± | 1 | 25 | ± | 2 |
|  | B-S 6 | 23 | 0.51 | ± | 0.04 | 0.49 | ± | 0.04 | 24 | ± | 3 | 0.86 | ± | 0.04 | 0.35 | ± | 0.04 | 7 | ± | 2 | 13 | ± | 2 | 44 | ± | 4 |
| Control group | Outcross | 142 | 0.25 | ± | 0.01 | 0.75 | ± | 0.01 | 28 | ± | 2 | 0.57 | ± | 0.02 | 0.16 | ± | 0.01 | 26 | ± | 2 | 5 | ± | 0 | 58 | ± | 3 |
|  | B-S 1 | 29 | 0.21 | ± | 0.02 | 0.79 | ± | 0.02 | 24 | ± | 2 | 0.63 | ± | 0.05 | 0.15 | ± | 0.03 | 17 | ± | 3 | 5 | ± | 1 | 45 | ± | 4 |
|  | B-S 2 | 18 | 0.15 | ± | 0.03 | 0.85 | ± | 0.03 | 18 | ± | 3 | 0.64 | ± | 0.05 | 0.22 | ± | 0.06 | 13 | ± | 4 | 3 | ± | 1 | 34 | ± | 4 |
|  | B-S 3 | 25 | 0.27 | ± | 0.04 | 0.73 | ± | 0.04 | 27 | ± | 3 | 0.76 | ± | 0.04 | 0.19 | ± | 0.04 | 9 | ± | 2 | 5 | ± | 1 | 41 | ± | 4 |
|  | B-S 4 | 5 | 0.27 | ± | 0.1 | 0.73 | ± | 0.1 | 18 | ± | 3 | 0.75 | ± | 0.02 | 0.13 | ± | 0.11 | 7 | ± | 1 | 2 | ± | 1 | 26 | ± | 3 |
|  | B-S 5 | 2 | 0.17 | ± | 0 | 0.83 | ± | 0 | 21 | ± | 15 | 0.72 | ± | 0.15 | 0.13 | ± | 0.13 | 6 | ± | 1 | 1 | ± | 1 | 28 | ± | 14 |
|  | B-S 6 | 0 | 0 | | | 0 | | | 0 | | | 0 | | | 0 | | | 0 | | | 0 | | | 0 | | |

**Table S6.** Pairwise comparison of sex ratio (proportion male), adult wasp proportion, and non-emerged fly pupae proportion between outcross and inbred generations of *L. clavipes* after GLMMs. Estimated marginal means (EMMs) of each generation (left column) is compared to the generation shown in the top row (* indicates significant difference, - indicates no difference based on Tukey test *P* < 0.05). Upper right half indicates the differences (± SE). B-S indicates brother-sister cross.

| Sex ratio (proportion male) | | | | | | | | | | | | | | | | | |  | |
| --- | --- | --- | --- | --- | --- | --- | --- | --- | --- | --- | --- | --- | --- | --- | --- | --- | --- | --- | --- |
| Generation | Outcross | B-S 1 | | | B-S 2 | | | B-S 3 | | | B-S 4 | | | B-S 5 | | | B-S 6 | | |
| Outcross |  | 0.11 | ± | 0.10 | -0.17 | ± | 0.11 | -0.47 | ± | 0.11 | -0.34 | ± | 0.12 | -0.59 | ± | 0.14 | -0.89 | ± | 0.13 |
| B-S 1 | - |  |  |  | -0.28 | ± | 0.10 | -0.59 | ± | 0.11 | -0.46 | ± | 0.11 | -0.71 | ± | 0.14 | -1.00 | ± | 0.13 |
| B-S 2 | - | - | | |  |  |  | -0.31 | ± | 0.08 | -0.18 | ± | 0.09 | -0.43 | ± | 0.12 | -0.72 | ± | 0.11 |
| B-S 3 | * | * | | | * | | |  |  |  | 0.13 | ± | 0.08 | -0.12 | ± | 0.12 | -0.41 | ± | 0.11 |
| B-S 4 | - | * | | | - | | | - | | |  |  |  | -0.25 | ± | 0.11 | -0.54 | ± | 0.10 |
| B-S 5 | * | * | | | * | | | - | | | - | | |  |  |  | -0.29 | ± | 0.13 |
| B-S 6 | * | * | | | * | | | * | | | * | | | - | | |  |  |  |
|  |  |  |  |  |  |  |  |  |  |  |  |  |  |  |  |  |  |  |  |
| Brood size | | | | | | | | | | | | | | | | | |  |  |
| Generation | Outcross | B-S 1 | | | B-S 2 | | | B-S 3 | | | B-S 4 | | | B-S 5 | | | B-S 6 | | |
| Outcross |  | 0.26 | ± | 0.13 | 0.09 | ± | 0.14 | 0.09 | ± | 0.14 | 0.30 | ± | 0.15 | 0.64 | ± | 0.19 | 0.32 | ± | 0.19 |
| B-S 1 | - |  |  |  | -0.17 | ± | 0.14 | -0.17 | ± | 0.14 | 0.03 | ± | 0.14 | 0.37 | ± | 0.18 | 0.06 | ± | 0.18 |
| B-S 2 | - | - | | |  |  |  | 0.00 | ± | 0.14 | 0.20 | ± | 0.15 | 0.54 | ± | 0.19 | 0.23 | ± | 0.19 |
| B-S 3 | - | - | | | - | | |  |  |  | 0.21 | ± | 0.14 | 0.55 | ± | 0.18 | 0.23 | ± | 0.19 |
| B-S 4 | - | - | | | - | | | - | | |  |  |  | 0.34 | ± | 0.17 | 0.02 | ± | 0.18 |
| B-S 5 | * | - | | | - | | | - | | | - | | |  |  |  | -0.32 | ± | 0.21 |
| B-S 6 | - | - | | | - | | | - | | | - | | | - | | |  |  |  |
|  |  |  |  |  |  |  |  |  |  |  |  |  |  |  |  |  |  |  |  |
| Adult wasp proportion | | | | | | | | | | | | | | | | | |  |  |
| Generation | Outcross | B-S 1 | | | B-S 2 | | | B-S 3 | | | B-S 4 | | | B-S 5 | | | B-S 6 | | |
| Outcross |  | -0.28 | ± | 0.07 | 0.04 | ± | 0.09 | -0.59 | ± | 0.10 | 0.40 | ± | 0.11 | -1.11 | ± | 0.20 | 0.04 | ± | 0.13 |
| B-S 1 | * |  |  |  | 0.32 | ± | 0.08 | -0.32 | ± | 0.09 | 0.68 | ± | 0.10 | -0.83 | ± | 0.19 | 0.32 | ± | 0.13 |
| B-S 2 | - | * | | |  |  |  | -0.64 | ± | 0.07 | 0.36 | ± | 0.08 | -1.15 | ± | 0.18 | 0.00 | ± | 0.11 |
| B-S 3 | * | * | | | * | | |  |  |  | 0.99 | ± | 0.09 | -0.52 | ± | 0.19 | 0.63 | ± | 0.12 |
| B-S 4 | * | * | | | * | | | * | | |  |  |  | -1.51 | ± | 0.18 | -0.36 | ± | 0.10 |
| B-S 5 | * | * | | | * | | | - | | | * | | |  |  |  | 1.15 | ± | 0.19 |
| B-S 6 | - | - | | | - | | | * | | | * | | | * | | |  |  |  |
|  |  |  |  |  |  |  |  |  |  |  |  |  |  |  |  |  |  |  |  |
| Non-emerged fly pupae proportion | | | | | | | | | | | | | | | | | |  |  |
| Generation | Outcross | B-S 1 | | | B-S 2 | | | B-S 3 | | | B-S 4 | | | B-S 5 | | | B-S 6 | | |
| Outcross |  | -1.20 | ± | 0.16 | -1.79 | ± | 0.18 | -2.18 | ± | 0.19 | -2.36 | ± | 0.19 | -2.56 | ± | 0.20 | -2.90 | ± | 0.19 |
| B-S 1 | * |  |  |  | -0.59 | ± | 0.14 | -0.98 | ± | 0.14 | -1.16 | ± | 0.14 | -1.36 | ± | 0.16 | -1.70 | ± | 0.15 |
| B-S 2 | * | * | | |  |  |  | -0.39 | ± | 0.10 | -0.58 | ± | 0.10 | -0.78 | ± | 0.13 | -1.11 | ± | 0.11 |
| B-S 3 | * | * | | | * | | |  |  |  | -0.18 | ± | 0.09 | -0.38 | ± | 0.12 | -0.72 | ± | 0.10 |
| B-S 4 | * | * | | | * | | | - | | |  |  |  | -0.20 | ± | 0.11 | -0.54 | ± | 0.09 |
| B-S 5 | * | * | | | * | | | * | | | - | | |  |  |  | -0.33 | ± | 0.12 |
| B-S 6 | * | * | | | * | | | * | | | * | | | - | | |  |  |  |


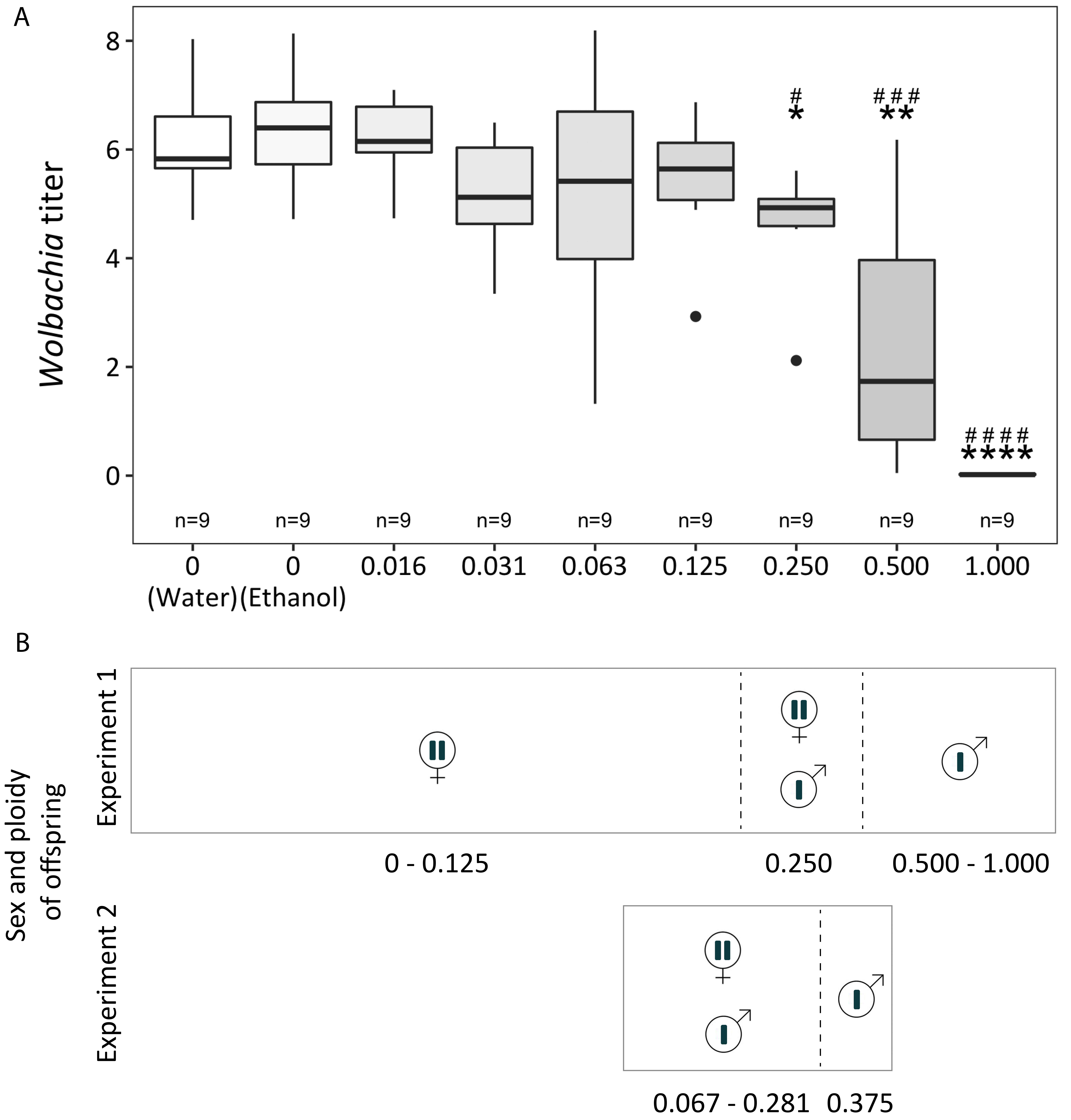


**Figure S3. A.** *Wolbachia* titer as function of various tetracycline concentrations. 0 (Water) and 0 (Ethanol) are control treatments with no tetracycline. Asterisks indicate significant differences (* P < 0.05, ** P < 0.01, *** P < 0.001, **** P < 0.0001) of experimental groups with 0 (Water) and hash tags (^#^ P < 0.05, ^##^ P < 0.01, ^###^ P < 0.001, ^####^ P < 0.0001) with 0 (Ethanol) control. B. Sex and ploidy of offspring in experiment 1 and 2. X axis indicates the range of antibiotic concentrations (tetracycline/yeast, mg/g).
