# Supplementary material for: Absence of complementary sex determination in two Leptopilina species (Figitidae, Hymenoptera) and a reconsideration of its incompatibility with endosymbiont‐induced thelytoky
Source: Insect Sci. 2021 Oct 25;29(3):900–14. doi: 10.1111/1744-7917.12969 (PMC9297927; doi:10.1111/1744-7917.12969)
Supplement: Supplementary file 2 — Supplementary Material [file INS-29-900-s002.docx]

/source_code/

Contains the source code in C++ for the simulation program. The program makes use of the datasets in

../input_data/, and generates the datasets in ../output_data/. Each separate script has been

documented using comments for ease of use. Simulation settings must be set using parameter.h. Within

parameter.h, some of the parameters used have only been used during development of the software and

are now effectively deprecated or simply non-functional; these are indicated as "DEPRECATED". Again,

comments provide further details on the code itself, but the following parameters are of particular

importance:

* n_loci = Control number of putative CSD loci in the simulation. We used 1/2/5/10 CSD loci in

different simulations.

* species = Controls the species from which the pedigree data is used to sample brood sizes and

fertilization rates. "cla" = L. clavipes, "het" = L. heterotoma. Note that we used

different values for the cross_type variable depending on species!

* cross_type = Controls whether the first inbreeding cross (F1 cross) is mother-son (M-S = 1) or

brother-sister (B-S = 0). We used M-S (setting 1) for L. heterotoma and B-S for L.

clavipes (setting 0).

--------------------------------------------------------------------------------------------------------------------------

Below is Cross.cpp

--------------------------------------------------------------------------------------------------------------------------

#include "Cross.h"

#include <iostream>

Cross::Cross(Individual Mom, Individual Dad, int nHaploid, int nDiploid)

{

// Generate nHaploid haploid sons.

for (size_t i = 0; i < nHaploid; ++i)

{

Individual HaploidSon(Mom.getGamete());

HaploidMale.push_back(HaploidSon);

// Print genotype to output, used for validation purposes so commented out.

// std::cout << i << "\t" << "HM" << "\t";

// HaploidSon.printHaplotypes();

}

// Generate nDiploid diploid offspring; determine sex and add to relevant "offspring groups" (diploid female or diploid male).

for (size_t i = 0; i < nDiploid; ++i)

{

Individual DiploidOffspring(Mom.getGamete(), Dad.getGamete());

DiploidOffspring.SexDetermination();

if (DiploidOffspring.isFemale)

{

DiploidFemale.push_back(DiploidOffspring);

// Print genotype to output, used for validation purposes so commented out.

// std::cout << i << "\t" << "DF" << "\t";

// DiploidOffspring.printHaplotypes();

}

else

{

if (ru() < diploid_male_survival)

{

DiploidMale.push_back(DiploidOffspring);

// Print genotype to output, used for validation purposes so commented out.

// std::cout << i << "\t" << "DM" << "\t";

// DiploidOffspring.printHaplotypes();

}

}

}

}

Cross::Cross(Individual Mom, int nHaploid, int nDiploid)

{

// Generate nHaploid haploid sons.

for (size_t i = 0; i < nHaploid; ++i)

{

Individual HaploidSon(Mom.getGamete());

HaploidMale.push_back(HaploidSon);

// Print genotype to output, used for validation purposes so commented out.

// std::cout << i << "\t" << "HM" << "\t";

// HaploidSon.printHaplotypes();

}

// Generate nDiploid diploid offspring; determine sex and add to relevant "offspring groups" (diploid female or diploid male).

for (size_t i = 0; i < nDiploid; ++i)

{

// !!! IMPORTANT !!!

// NO ACTUAL SON IS SIMULATED! INSTEAD WE SIMULATE THAT THE MOM REPRODUCES BY PRODUCING BOTH GAMETES!

// BEAR IN MIND THAT THE SON IS BASICALLY A GAMETE FROM THE MOTHER AND THEREFORE PURELY A TEMPORARY VECTOR IN THIS TYPE OF CROSS!

Individual DiploidOffspring(Mom.getGamete(), Mom.getGamete());

DiploidOffspring.SexDetermination();

if (DiploidOffspring.isFemale)

{

DiploidFemale.push_back(DiploidOffspring);

// Print genotype to output, used for validation purposes so commented out.

// std::cout << i << "\t" << "DF" << "\t";

// DiploidOffspring.printHaplotypes();

}

else

{

if (ru() < diploid_male_survival)

{

DiploidMale.push_back(DiploidOffspring);

// Print genotype to output, used for validation purposes so commented out.

// std::cout << i << "\t" << "DM" << "\t";

// DiploidOffspring.printHaplotypes();

}

}

}

}

void Cross::writeResults(std::ofstream &results, int replicate, int generation)

{

results << n_loci << "\t";

results << replicate << "\t";

results << generation << "\t";

results << HaploidMale.size() << "\t";

results << DiploidMale.size() << "\t";

results << DiploidFemale.size() << "\t";

results << std::endl;

}

--------------------------------------------------------------------------------------------------------------------------

Below is Cross.h

--------------------------------------------------------------------------------------------------------------------------

#pragma once

#include "Individual.h"

#include "randomnumbers.h"

#include <vector>

#include <fstream>

class Cross

{

public:

// constructors

Cross() = default;

Cross(Individual Mom, Individual Dad, int nHaploid, int nDiploid); // Brother-sister cross function

Cross(Individual Mom, int nHaploid, int nDiploid); // Mother-son cross function

// traits

std::vector<Individual> HaploidMale;

std::vector<Individual> DiploidMale;

std::vector<Individual> DiploidFemale;

// functions

void writeResults(std::ofstream &results, int replicate, int generation);

};

--------------------------------------------------------------------------------------------------------------------------

Below is Family.cpp

--------------------------------------------------------------------------------------------------------------------------

#include "Family.h"

#include "Cross.h"

#include "utils.h"

#include <array>

#include <iostream>

Family::Family()

{

Individual Patriarch(false);

Individual Matriarch(true);

Dads.push_back(Patriarch);

Moms.push_back(Matriarch);

}

void Family::outcross(std::ofstream &results, int replicate, std::vector< std::vector< int > > offsprings)

{

// Empty vectors for new brother-sister pairs.

SelectedDaughters.clear();

SelectedSons.clear();

// Write function to sample haploid + diploid numbers from value in dataset.

std::array<int, 2 > offspring = sample_data_file(offsprings, Generation);

// Have brothers (dads) and sisters (moms) make babies.

Cross Cross(Moms[0], Dads[0], offspring[1], offspring[0]);

// Write current generation + counts of haploid male, diploid male & diploid female offspring to results

Cross.writeResults(results, replicate, Generation);

// From haploid males and diploid females, sample up to n_offspring_pairs_used brother-sister pairs for next generation.

int j = 0;

while (j < n_offspring_pairs_used & Cross.DiploidFemale.size() > 0 & Cross.HaploidMale.size() > 0)

{

// Set up discrete distributions to sample offspring, pretty ugly. Ideally this should be outside

// this while-statement, but then you need to cut out elements from the discrete_distribution. That's

// too much of a hassle to write for now. Instead, I just re-make the distro's every time.

std::vector<double> M;

std::vector<double> F;

for (int i = 0; i < Cross.HaploidMale.size(); ++i) { M.push_back(1.0); }

for (int i = 0; i < Cross.DiploidFemale.size(); ++i) { F.push_back(1.0); }

std::discrete_distribution<> sampleM(M.begin(), M.end());

std::discrete_distribution<> sampleF(F.begin(), F.end());

// Sample new dads + moms.

int dadID = sampleM(rng);

int momID = sampleF(rng);

// Store them in 'selected' vectors.

SelectedSons.push_back(Cross.HaploidMale[dadID]);

SelectedDaughters.push_back(Cross.DiploidFemale[momID]);

// Remove them from the pool of individuals (SAMPLE WITHOUT REPLACEMENT!)

Cross.HaploidMale.erase(Cross.HaploidMale.begin() + dadID);

Cross.DiploidFemale.erase(Cross.DiploidFemale.begin() + momID);

++j;

}

swap(SelectedDaughters, Moms);

swap(SelectedSons, Dads);

++Generation;

}

void Family::reproduceBS(std::ofstream &results, int replicate, std::vector< std::vector< int > > offsprings)

{

// Empty vectors for new brother-sister pairs.

SelectedDaughters.clear();

SelectedSons.clear();

for (int i = 0; i < Moms.size(); ++i)

{

// Write function to sample haploid + diploid numbers from value in dataset.

std::array<int, 2 > offspring = sample_data_file(offsprings, Generation);

// Have brothers (dads) and sisters (moms) make babies.

Cross Cross(Moms[i], Dads[i], offspring[1], offspring[0]);

// Write current generation + counts of haploid male, diploid male & diploid female offspring to results

Cross.writeResults(results, replicate, Generation);

// From haploid males and diploid females, sample up to n_offspring_pairs_used brother-sister pairs for next generation.

int j = 0;

while (j < n_offspring_pairs_used & Cross.DiploidFemale.size() > 0 & Cross.HaploidMale.size() > 0)

{

// Set up discrete distributions to sample offspring, pretty ugly. Ideally this should be outside

// this while-statement, but then you need to cut out elements from the discrete_distribution. That's

// too much of a hassle to write for now. Instead, I just re-make the distro's every time.

std::vector<double> M;

std::vector<double> F;

for (int i = 0; i < Cross.HaploidMale.size(); ++i) { M.push_back(1.0); }

for (int i = 0; i < Cross.DiploidFemale.size(); ++i) { F.push_back(1.0); }

std::discrete_distribution<> sampleM(M.begin(), M.end());

std::discrete_distribution<> sampleF(F.begin(), F.end());

// Sample new dads + moms.

int dadID = sampleM(rng);

int momID = sampleF(rng);

// Store them in 'selected' vectors.

SelectedSons.push_back(Cross.HaploidMale[dadID]);

SelectedDaughters.push_back(Cross.DiploidFemale[momID]);

// Remove them from the pool of individuals (SAMPLE WITHOUT REPLACEMENT!)

Cross.HaploidMale.erase(Cross.HaploidMale.begin() + dadID);

Cross.DiploidFemale.erase(Cross.DiploidFemale.begin() + momID);

++j;

}

}

swap(SelectedDaughters, Moms);

swap(SelectedSons, Dads);

++Generation;

}

void Family::reproduceMS(std::ofstream &results, int replicate, std::vector< std::vector< int > > offsprings)

{

// Empty vectors for new brother-sister pairs.

SelectedDaughters.clear();

SelectedSons.clear();

for (int i = 0; i < Moms.size(); ++i)

{

// Write function to sample haploid + diploid numbers from value in dataset.

std::array<int, 2 > offspring = sample_data_file(offsprings, Generation);

// Have moms babies (sons are not simulated but simply 'sons' are sampled as gametes from the mother on-the-go.

Cross Cross(Moms[i], offspring[1], offspring[0]);

// Write current generation + counts of haploid male, diploid male & diploid female offspring to results

Cross.writeResults(results, replicate, Generation);

// From haploid males and diploid females, sample up to n_offspring_pairs_used brother-sister pairs for next generation.

int j = 0;

while (j < n_offspring_pairs_used & Cross.DiploidFemale.size() > 0 & Cross.HaploidMale.size() > 0)

{

// Set up discrete distributions to sample offspring, pretty ugly. Ideally this should be outside

// this while-statement, but then you need to cut out elements from the discrete_distribution. That's

// too much of a hassle to write for now. Instead, I just re-make the distro's every time.

std::vector<double> M;

std::vector<double> F;

for (int i = 0; i < Cross.HaploidMale.size(); ++i) { M.push_back(1.0); }

for (int i = 0; i < Cross.DiploidFemale.size(); ++i) { F.push_back(1.0); }

std::discrete_distribution<> sampleM(M.begin(), M.end());

std::discrete_distribution<> sampleF(F.begin(), F.end());

// Sample new dads + moms.

int dadID = sampleM(rng);

int momID = sampleF(rng);

// Store them in 'selected' vectors.

SelectedSons.push_back(Cross.HaploidMale[dadID]);

SelectedDaughters.push_back(Cross.DiploidFemale[momID]);

// Remove them from the pool of individuals (SAMPLE WITHOUT REPLACEMENT!)

Cross.HaploidMale.erase(Cross.HaploidMale.begin() + dadID);

Cross.DiploidFemale.erase(Cross.DiploidFemale.begin() + momID);

++j;

}

}

swap(SelectedDaughters, Moms);

swap(SelectedSons, Dads);

++Generation;

}

--------------------------------------------------------------------------------------------------------------------------

Below is Family.h

#pragma once

#include "Individual.h"

#include "Cross.h"

#include <algorithm>

#include "randomnumbers.h"

class Family

{

public:

// constructor

Family();

// traits

int Generation = 0;

std::vector<Individual> Moms;

std::vector<Individual> Dads;

std::vector<Individual> SelectedDaughters;

std::vector<Individual> SelectedSons;

// functions

void outcross(std::ofstream &results, int replicate, std::vector< std::vector< int > > offsprings);

void reproduceBS(std::ofstream &results, int replicate, std::vector< std::vector< int > > offsprings);

void reproduceMS(std::ofstream &results, int replicate, std::vector< std::vector< int > > offsprings);

};

--------------------------------------------------------------------------------------------------------------------------

Below is Haplotype.cpp

#include "Haplotype.h"

// Write alleles to a haplotype.

Haplotype::Haplotype(std::array<int, n_loci> setHaplo)

{

for (int i = 0; i < n_loci; ++i)

{

Alleles[i] = setHaplo[i];

}

}

// Initialize haplotype with all loci have a given locus-specific allele (all loci 0, 1, etc.)

// Because alleles don't switch loci, we can have allele 0 at all loci, e.g. a haplotype 0000.

Haplotype::Haplotype(int setInitialHaplotype)

{

for (size_t i = 0; i < n_loci; ++i)

{

Alleles[i] = setInitialHaplotype;

}

}

// Retrieve the entire haplotype in one go.

std::array<int, n_loci> Haplotype::getHaplotype()

{

return Alleles;

}

// Retrieve a single allele from a haplotype.

int Haplotype::sampleAllele(int Locus)

{

return Alleles[Locus];

}

--------------------------------------------------------------------------------------------------------------------------

Below is Haplotype.h

#pragma once

#include "parameters.h"

#include <array>

#include <algorithm>

#include <optional>

class Haplotype {

public:

// constructors

Haplotype() = default;

Haplotype(std::array<int, n_loci> setHaplo);

Haplotype(int setInitialHaplotype);

// traits

std::array<int, n_loci> Alleles = { 0 };

// functions

std::array<int, n_loci> getHaplotype();

int sampleAllele(int Locus);

};

--------------------------------------------------------------------------------------------------------------------------

Below is Individual.cpp

#include "Individual.h"

#include "randomnumbers.h"

#include "parameters.h"

#include <iostream>

// Generator for simulating initial outcross

Individual::Individual(bool isGirl)

{

if (isGirl)

{

Maternal = Haplotype(0);

Paternal = Haplotype(1);

Ploidy = 2;

}

else

{

Maternal = Haplotype(2);

}

isFemale = isGirl;

}

// Generator for making haploid individuals from sampled gametes (everything except initialisation).

Individual::Individual(Haplotype fromMom)

{

Maternal = fromMom;

}

// Generator for making diploid individuals from sampled gametes (everything except initialisation).

Individual::Individual(Haplotype fromMom, Haplotype fromDad)

{

Maternal = fromMom;

Paternal = fromDad;

Ploidy = 2;

}

// Sex determination function.

void Individual::SexDetermination()

{

if(Ploidy == 2)

{

// Sequentially check all loci until 1 is heterozygous. If yes, then female.

bool Heterozygote = false;

size_t i = 0;

while (i < n_loci & Heterozygote == false)

{

if (Paternal.getHaplotype()[i] != Maternal.getHaplotype()[i])

{

Heterozygote = true;

isFemale = true;

}

++i;

}

}

}

// Generate a gamete from an individual's haplotype(s).

Haplotype Individual::getGamete() {

// If haploid, just pass on the maternal (=only) haplotype.

if (Ploidy == 1)

{

return(Maternal);

}

// if diploid, sample alleles from maternal/paternal haplotype.

else {

std::array<int, n_loci> haplo;

// If loci are linked, sample alleles with possibly recombination between successive loci.

if (linked)

{

bool previousPaternal = r2();

if (previousPaternal)

{

haplo[0] = Paternal.sampleAllele(0);

}

for (int i = 1; i < n_loci; ++i)

{

// If allele at previous locus was sampled from the paternal, determine

// if next is from maternal (recombination) or paternal (no recombination)

if (previousPaternal)

{

// Test if recombination occurs

if (r2(recombination[i - 1]))

{

// If yes, then sample maternal allele and update previously-sampled allele status

haplo[i] = Maternal.sampleAllele(i);

previousPaternal = 0;

}

else

{

// If not, just sample the paternal allele.

haplo[i] = Paternal.sampleAllele(i);

}

}

else {

// This is the same function as the above, but then assuming the previous allele was sampled

// from the maternal haplotype.

if (r2(recombination[i - 1]))

{

haplo[i] = Paternal.sampleAllele(i);

previousPaternal = 1;

}

else

{

haplo[i] = Maternal.sampleAllele(i);

}

}

Haplotype Gamete(haplo);

return Gamete;

}

}

// If loci are unlinked, just randomly sample maternal/paternal alleles.

else

{

for (int i = 0; i < n_loci; ++i)

{

// Randomly sample maternal (r2() = 0) or paternal (r2() = 1) allele

if(r2())

{

haplo[i] = Paternal.sampleAllele(i);

}

else

{

haplo[i] = Maternal.sampleAllele(i);

}

}

Haplotype Gamete(haplo);

return Gamete;

}

}

}

void Individual::printHaplotypes()

{

if (Ploidy == 1)

{

// Print maternal haplotype.

for (int i = 0; i < n_loci; ++i)

{

std::cout << Maternal.Alleles[i];

}

std::cout << std::endl;

}

else {

// Print maternal haplotype.

for (int i = 0; i < n_loci; ++i)

{

std::cout << Maternal.Alleles[i];

}

// Print paternal haplotype.

for (int i = 0; i < n_loci; ++i)

{

std::cout << Paternal.Alleles[i];

}

std::cout << std::endl;

}

}

--------------------------------------------------------------------------------------------------------------------------

Below is Individual.h

--------------------------------------------------------------------------------------------------------------------------

#pragma once

#include "Haplotype.h"

class Individual {

public:

// constructors

Individual(bool isGirl); // Initialisation

Individual(Haplotype fromMom); // Haploid individuals (= sons) from gamete

Individual(Haplotype fromMom, Haplotype fromDad); // diploid individuals from gametes

// traits

Haplotype Maternal;

Haplotype Paternal;

int Ploidy = 1;

bool isFemale = 0; // Only female if paternal haplotype is present and at least 1 locus is heterozygous

// functions

Haplotype getGamete();

void printHaplotypes();

void SexDetermination();

};

--------------------------------------------------------------------------------------------------------------------------

Below is main.cpp

--------------------------------------------------------------------------------------------------------------------------

#include <array>

#include <iostream>

#include "Family.h"

#include <optional>

#include <fstream>

#include <string>

#include <vector>

#include "utils.h"

#include <algorithm>

int main()

{

// SET UP SIMULATION //

long seed = randomize(); // Set seed for RNG

std::vector< std::vector< int > > data; // Read data

int max_gen;

read_data_file(data_file, data, max_gen);

std::cout << "Simulating " << n_replicates << " replicates with " << max_gen << " generations..." << std::endl << std::endl;

std::ofstream results("2021_01_04_BS_n_" + std::to_string(n_loci) + "_" + species + ".txt");

results << "n_loci" << "\t" << "replicate" << "\t" << "generation" << "\t" << "haploid_male" << "\t" << "diploid_male" << "\t" << "diploid_female" << std::endl;

for (int n = 0; n < n_replicates; ++n)

{

// Set up outcross

Family tFamily;

tFamily.outcross(results, n, data);

// Perform first cross after outcross, if cross_type = true use mother-son

if (cross_type)

{

tFamily.reproduceMS(results, n, data);

}

// otherwise use brother-sister cross

else {

tFamily.reproduceBS(results, n, data);

}

// Run remainder of experiment.

for (int t = 1; t < max_gen; ++t)

{

tFamily.reproduceBS(results, n, data);

}

if (n % 100 == 0)

{

std::cout << n << "/" << n_replicates << " simulations done. " << std::endl;

}

}

return 0;

}

--------------------------------------------------------------------------------------------------------------------------

Below is parameters.h

--------------------------------------------------------------------------------------------------------------------------

#pragma once

#include <array>

// Basic simulations parameters:

const int n_generations = 10; // Number of generations to simulate AFTER outcross. DEPRECATED, used for development but now set by data from pedigree used.

const int n_replicates = 10000; // Number of replicate crosses to simulate.

const int n_offspring_pairs_used = 1; // Number of daughters that are used every generation. Keep to 1 or simulation will grow exponentially. Only used for development.

const int n_loci = 10; // Number of CSD loci considered.

const std::string species = "cla"; // species to be tested, cla = clavipes, het = heterotoma

const std::string data_file = "test_input_from_" + species + ".csv"; //

// Recombination parameters

const bool linked = false; // If true, provide a vector of n-1 recombination rates (or 1 constant for all rates).

const std::array<double, n_loci - 1> recombination = { 0.5 }; // Recombination rate between loci.

// F1 cross type (brother-sister = 0, mother-son = 1)

// Note that F0 is always assumed to be an outcross.

const bool cross_type = 0;

// Diploid male survival rate (0-1)

const double diploid_male_survival = 1.0;

// Family size parameters - DEPRECATED, only used for development.

const int haploids = 10; // Number of haploid offspring generated in cross.

const int diploids = 50; // Number of diploid offspring generated in cross.

// Sampling methodology

// true = samples brood sizes and fertilization rates from females in the matching generation in the experiment.

// false = samplesbrood sizes and fertilization rates from females in any generation in the experiment.

const bool sample_current_generation = false;

--------------------------------------------------------------------------------------------------------------------------

Below is randomnumbers.cpp

--------------------------------------------------------------------------------------------------------------------------

#include "randomnumbers.h"

std::mt19937 rng;

// set up seed

long randomize() {

static std::random_device rd{};

auto seed = rd();

rng.seed(seed);

return seed;

}

// random double [0,1)

double ru()

{

std::uniform_real_distribution<> d{};

return d(rng);

}

// random standard normal

double rnorm(const double &mean, const double &stddev)

{

std::normal_distribution<> d{mean, stddev};

return d(rng);

}

// random bernoulli {0,1}

bool r2(const double& p)

{

static std::bernoulli_distribution d{};

using parm_t = decltype(d)::param_type;

return d(rng, parm_t{p});

}

int rpois(const double &lambda)

{

std::poisson_distribution<> d{};

using parm_t = decltype(d)::param_type;

return d(rng, parm_t{lambda});

}

double rexp(const double &lambda)

{

std::exponential_distribution<> d{};

using parm_t = decltype(d)::param_type;

return d(rng, parm_t{ lambda });

}

--------------------------------------------------------------------------------------------------------------------------

Below is randomnumbers.h

--------------------------------------------------------------------------------------------------------------------------

#ifndef RANDOMNUMBERS_H

#define RANDOMNUMBERS_H

#include <random>

long randomize();

extern std::mt19937 rng;

// random integer [0,n)

template <typename T>

T rn(const T n){

static std::uniform_int_distribution<T> d{0,n-1};

return d(rng);

}

// random uniform [0,1)

double ru();

// random standard normal

double rnorm(const double &mean, const double &stddev);

// random binary

bool r2(const double& p = 0.5);

// random Poisson

int rpois(const double&);

// random exponential

double rexp(const double&);

extern std::mt19937 rng;

template <typename T1 = double, typename T2 = size_t>

class ridx{

public:

ridx(const std::vector<T1>& w) : w(w) {}

T2 operator()() const {

static std::discrete_distribution<T2> d(w.begin(),w.end());

return d(rng);

}

private:

const std::vector<T1>& w;

};

#endif // RANDOMNUMBERS_H

--------------------------------------------------------------------------------------------------------------------------

Below is utils.cpp

--------------------------------------------------------------------------------------------------------------------------

#include <fstream>

#include <sstream>

#include <iomanip>

#include <chrono>

#include <ctime>

#include <array>

#include <cmath>

#include <cassert>

#include <iostream>

#include <numeric>

#include <vector>

#include "parameters.h"

#include "randomnumbers.h"

#include "utils.h"

#define _CRT_SECURE_NO_WARNINGS

bool read_data_file(std::string file, std::vector< std::vector< int > > &df, int &max_gen)

{

std::ifstream in(file);

if (!in)

{

std::cerr << "ERROR: BAD FILE!\n";

return false;

};

std::string line;

max_gen = 0;

int n_lines = 0;

while (std::getline(in, line))

{

if ((n_lines == 0))

{

std::stringstream stream(line);

std::string cell;

std::vector< std::string > row;

std::cout << "Reading variables: " << std::endl;

while (std::getline(stream, cell, ';'))

{

std::cout << "* "<< cell << std::endl;

}

std::cout << std::endl;

++n_lines;

}

else {

std::stringstream stream(line);

std::string cell;

std::vector< int > row;

while (std::getline(stream, cell, ';'))

{

row.push_back(stoi(cell));

}

// Update max_gen

max_gen < row[0] ? max_gen = row[0] : max_gen = max_gen;

df.push_back(row);

++n_lines;

}

};

return true;

}

std::array<int, 2> sample_data_file(std::vector< std::vector< int > > df, int generation)

{

std::vector < std::vector < int > > df_to_sample;

// Assess which generation(s) must be used

if (sample_current_generation)

{

// Extract matching rows.

for (int i = 0; i < df.size(); ++i)

{

if (df[i][0] == generation)

{

std::vector<int> row = { df[i][0], df[i][1], df[i][2] };

df_to_sample.push_back(row);

}

}

}

else {

df_to_sample = df;

}

// Count number of entries N

int no_entries = df_to_sample.size();

// Generate vector of N times 1.0

std::vector<double> uniform_probabilities;

for (int i = 0; i < no_entries; ++i)

{

uniform_probabilities.push_back(1.0);

}

// Convert to discrete distribution

std::discrete_distribution<> sampleID(uniform_probabilities.begin(), uniform_probabilities.end());

// Sample entry

int sampled_row = sampleID(rng);

//std::cout << sampled_row << "\t" << df_to_sample[sampled_row][0] << "\t" << df_to_sample[sampled_row][1] << "\t" << df_to_sample[sampled_row][2];

std::array<int, 2> sampled = { df_to_sample[sampled_row][1], df_to_sample[sampled_row][2]};

return(sampled);

}

--------------------------------------------------------------------------------------------------------------------------

Below is utils.h

--------------------------------------------------------------------------------------------------------------------------

#include <string>

#include <vector>

#include <array>

bool read_data_file(std::string file, std::vector< std::vector< int > > &df, int &max_gen);

std::array<int, 2> sample_data_file(std::vector< std::vector< int > > df, int generation);
